# FuTCM-PDD: a multi-level information fusion framework integrating KAN modeling and AutoML for phenotype-based drug discovery from Traditional Chinese Medicine

**DOI:** 10.1186/s13020-026-01490-1

**Published:** 2026-09-21

**Authors:** Zewen Wang, Yanxia Liu, Yue Ren, Qun Li, Jiaye Tian, Bin Yu, Yuezhong Zhu, Jiaqi Wang, Miao Li, Liansheng Qiao, Yanling Zhang

**Affiliations:** Key Laboratory of TCM-Information Engineer of State Administration of TCM, School of Chinese Materia Medica, Beijing University of Chinese Medicine, Beijing, 102488 China

**Keywords:** Information fusion, Kolmogorov–Arnold network, Traditional Chinese Medicine, AutoML, Phenotype-based drug discovery

## Abstract

Phenotype-based drug discovery has attracted increasing attention due to its critical role in first-in-class drug development and higher clinical translation rates. Chinese Materia Medica (CMM), with its emphasis on properties and efficacies, provides a unique framework for phenotype-oriented therapeutic discovery. We developed an AI-driven framework, FuTCM-PDD, that integrates CMMs, compounds, targets, pathways, pharmacological effects, CMM efficacies, and CMM properties to enable phenotype-based drug discovery. For drug-target interaction (DTI) prediction, a Kolmogorov–Arnold Network (KAN) model with circle loss optimization was established, outperforming the best baseline by 16.14% in Recall@10 and validated by literature, databases, and molecular docking. In addition, an Automated Machine Learning (AutoML) framework integrating 168 machine learning algorithms was implemented using pharmacological effects to predict CMM properties and efficacies. By integrating model predictions of targets, pharmacological effects, properties, and efficacies, covering 809,645 associations across 7141 CMM herbs, 30,731 compounds, 4321 targets, 40 pharmacological effects, 18 properties, and 18 efficacies, we constructed a multi-layer biological network centered on CMM properties and efficacies. The integrative network was validated via randomization analyses, establishing its biological significance and enabling quantitative mappings across CMM herbs, compounds, targets, pharmacological effects, and CMM phenotypic concepts through network-topology metrics, information-flow modeling, and randomized perturbation embedding. Using an approach distinct from conventional network pharmacology, the FuTCM-PDD identified a lipid-lowering pharmacological effect of *Notopterygii Rhizoma et Radix* (NRR, Qianghuo) and its active compounds, which were experimentally validated using an in vitro hepatic steatosis model. FuTCM-PDD provides an AI-assisted framework for phenotype-based discovery of potential drug candidates from CMM through multi-level data fusion, helping move beyond the recurrent prioritization of commonly reported associations in conventional network pharmacology and supporting the identification of pharmacologically relevant candidate compounds associated with specific phenotypic contexts.

**Graphical Abstract:**

**Figure:**
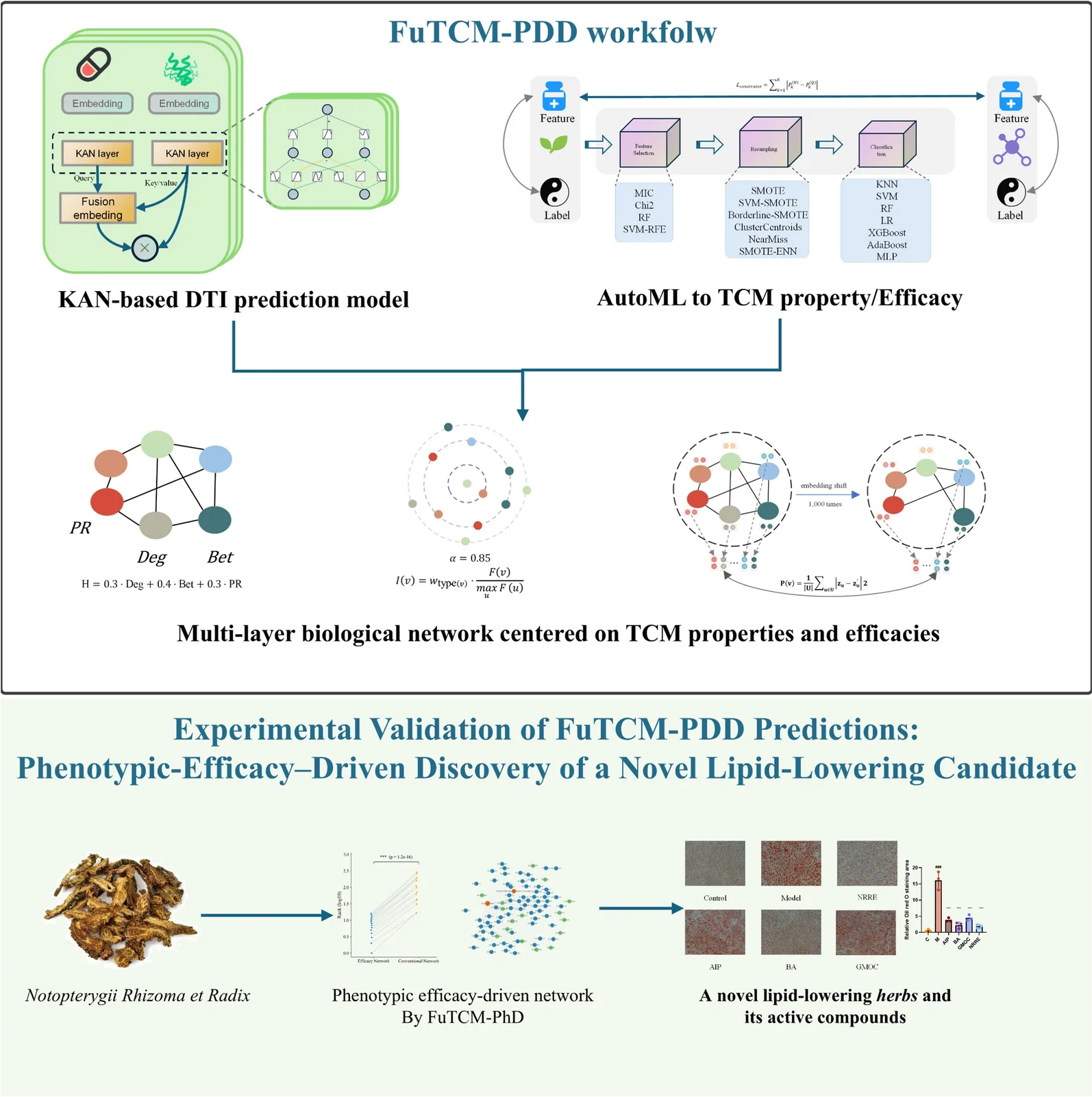

**Supplementary Information:**

The online version contains supplementary material available at https://doi.org/10.1186/s13020-026-01490-1.

## Introduction

Current strategies in drug discovery primarily include target-based and phenotype-based approaches [1]. Target-based drug discovery focuses on designing or screening compounds against specific protein targets, but it has faced growing limitations, including off-target effects, high costs, and low approval rates [2]. In contrast, phenotype-based drug discovery proceeds in the opposite direction: it first identifies compounds capable of reversing disease phenotypes and subsequently elucidates the underlying molecular mechanisms. This approach has regained prominence for its ability to yield first-in-class drugs [3], largely because it does not require prior knowledge of specific targets and often demonstrates higher clinical translation success rates [4]. However, despite these advantages, practical and systematic methodological frameworks for phenotype-based discovery are still lacking, which substantially limits its application in modern pharmacology.

Chinese Materia Medica (CMM) carries thousands of years of therapeutic knowledge. Centuries of clinical practice in CMM can be regarded as a form of natural phenotypic screening, in which CMM herbs are categorized by the CMM theory, such as properties and efficacies. These classifications capture consistent physiological responses and exhibit natural correlations with modern pharmacological effects. Recent studies have increasingly demonstrated the pharmaceutical potential of CMM, with numerous bioactive compounds—such as artemisinin [5], ephedrine [6], and paclitaxel [7]—successfully isolated from natural products. These examples underscore the potential of CMM to contribute valuable insights to phenotype-based drug discovery.

Nevertheless, translating the metaphorical and conceptual CMM theory into quantitative, target- and pathway-level mechanisms remains a major methodological challenge. Network pharmacology combined with experimental validation has begun to provide mechanistic insights into CMM theories [8, 9]. Representative network pharmacology–based implementations include SymMap, which links traditional symptom descriptions to their counterparts in modern medicine, and ETCM, which employs a network pharmacology framework to elucidate herb–symptom associations in TCM [10, 11]. Furthermore, TCMSSD applies an LSTM-based framework for syndrome standardization, thereby providing partial mechanistic interpretations of classical theories [12]. However, conventional network pharmacology suffers from substantial homogeneity, frequently identifying common compounds such as quercetin acting on widely shared targets like IL6 [13]. Methodologically, this homogeneity is largely driven by two factors. First, most current CMM-related target-prediction tools still rely on traditional machine-learning models [14] or simple similarity-based strategies [15], which provide limited feature representation and generalization for structurally diverse and novel CMM compounds, resulting in suboptimal accuracy. Second, the networks are usually restricted to the compound–target layer and loosely coupled to higher-level CMM concepts, leading to insufficient integration of CMM properties and efficacies with modern pharmacological effects.

**Fig. 1:**
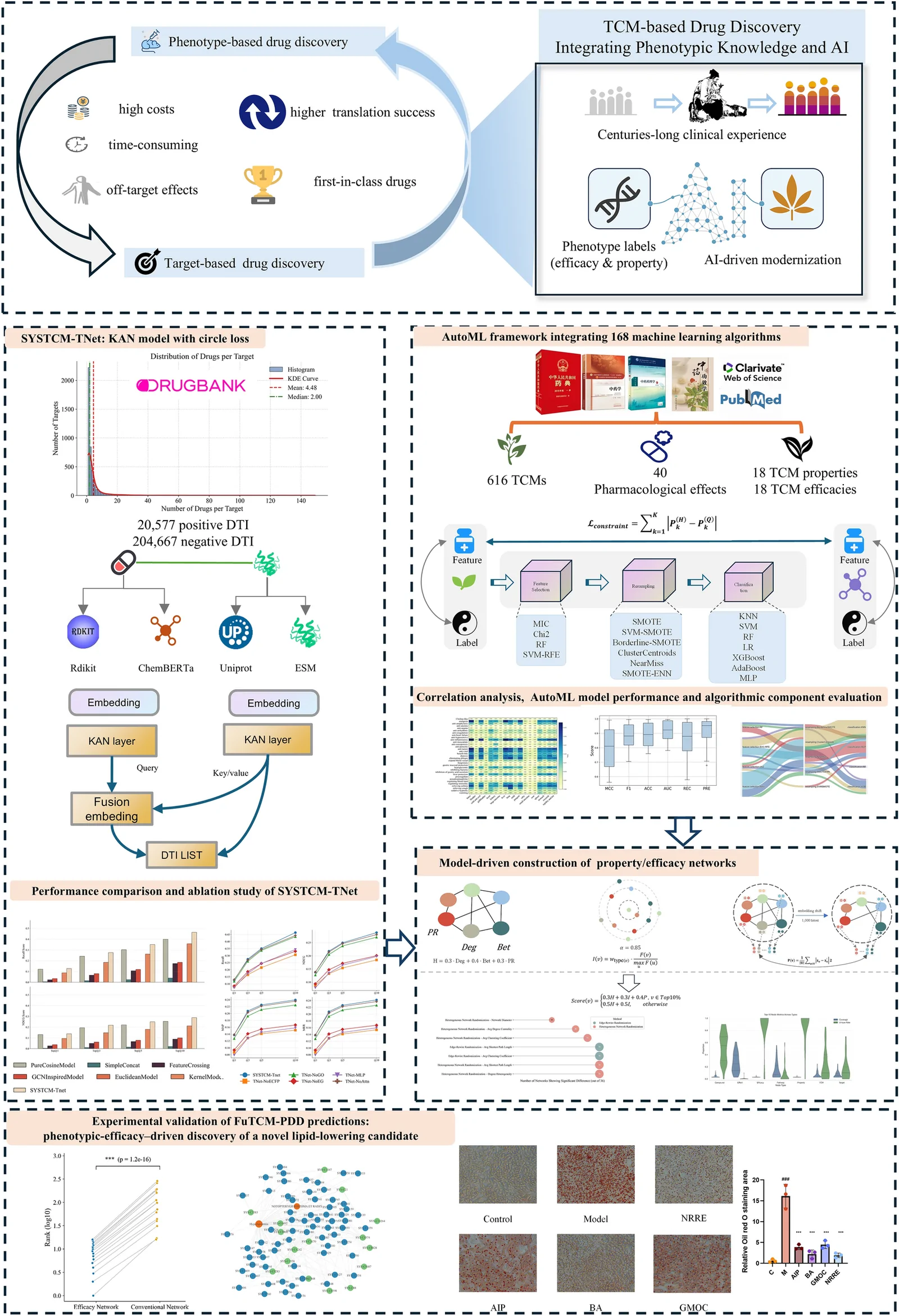
FuTCM-PDD: A multi-level information fusion framework bridging CMM theory and AI for phenotype-based drug discovery

To address these challenges, we developed FuTCM-PDD, the first AI- and machine learning–driven framework designed to comprehensively decode the properties and efficacies of CMMs for phenotype-based drug discovery (Fig. 1). As a core component, we constructed a Systematic Deep Learning Network Framework for Traditional Chinese Medicine Target Prediction (SYSTCM-TNet) based on the KAN algorithm. Compared to the baseline, SYSTCM-TNet provides richer nonlinear feature modeling for CMM compounds and substantially improves target-prediction performance, thereby offering a more reliable basis for downstream network pharmacology. In addition, FuTCM-PDD implements an OPTUNA-based AutoML framework that integrates 168 machine-learning algorithms and ultimately constructs 36 optimized predictive models to evaluate CMM properties and efficacies in relation to pharmacological effects. This automated and large-scale model selection strategy goes beyond conventional pipelines and yields more accurate and robust predictions of CMM attributes at the compound level. Building on these methodological advances, FuTCM-PDD constructs a multi-level integrative network by integrating 809,645 relationships among 7141 CMM herbs, 30,731 compounds, 4321 targets, 580 pathways, 40 pharmacological effects, 18 properties, and 18 efficacies. This multi-layer architecture enables systematic delineation of the correlations between CMMs, compounds, targets, pathways, properties, and efficacies within a unified network-pharmacology context and effectively alleviates the homogeneity of conventional networks dominated by a few common compounds and targets. As a case study, using the FuTCM-PDD framework with a phenotypic-efficacy–driven discovery strategy, we identified a novel lipid-lowering efficacy of NRR and its key active compounds, which were uncovered beyond the reach of conventional network pharmacology methods.

In summary, FuTCM-PDD establishes a phenotype-driven framework grounded in TCM knowledge that introduces a high-accuracy deep-learning architecture for CMM compound–target prediction, leverages an AutoML-based strategy to systematically model CMM properties and efficacies, and integrates these components into a multi-level network that bridges CMM theory with modern biomedicine. These methodological developments provide a novel computational perspective for phenotype-oriented network pharmacology and offer a systematic strategy for prioritizing potential pharmacological effects and candidate active compounds from CMM.

## Methods

### Data collection and preprocessing of DTI

**Fig. 2:**
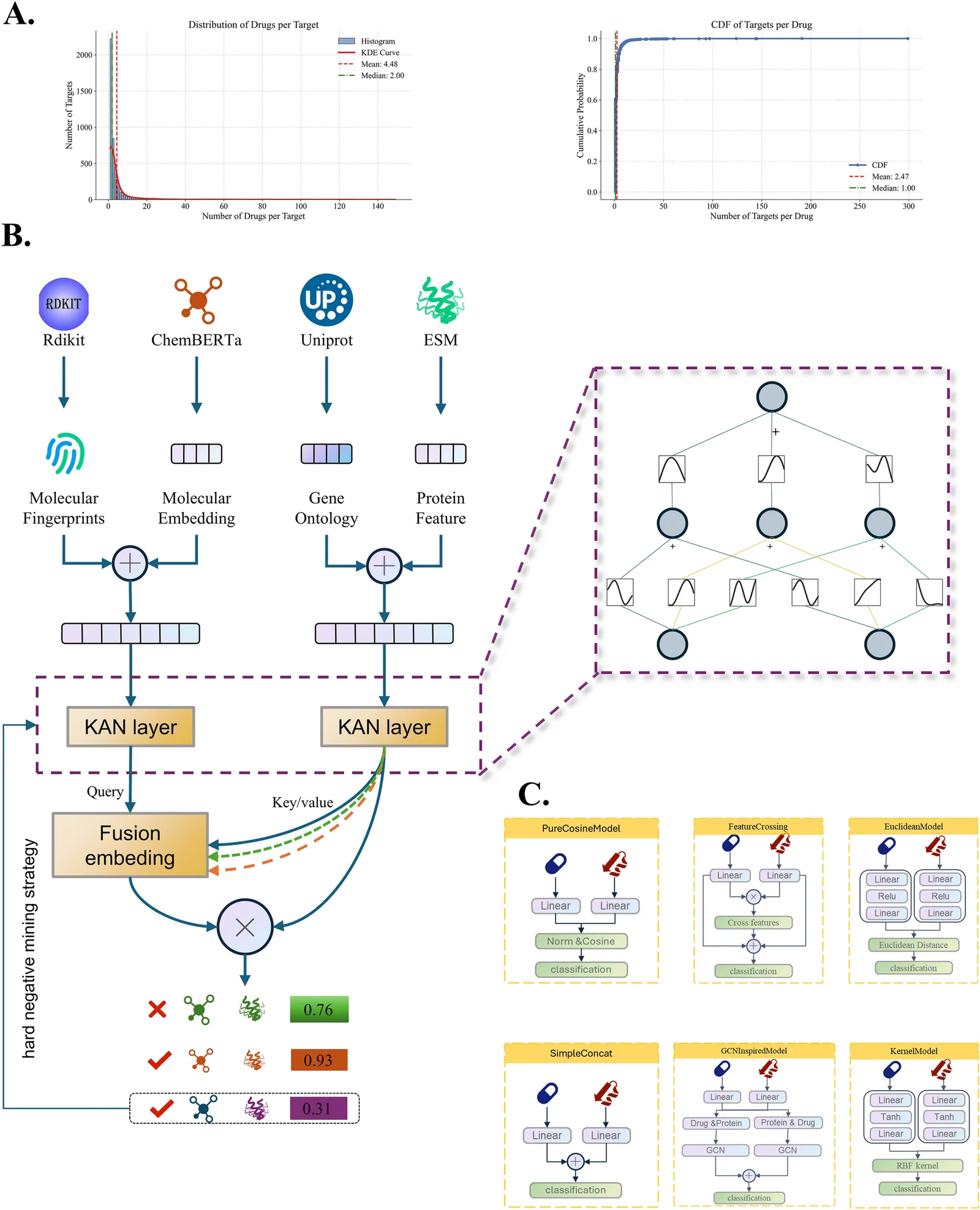
Dataset statistics and model framework. **A** Dataset description of SYSTCM-TNet. **B** Overall architecture of SYSTCM-TNet. **C** Baseline models: Cosine, FeatureCrossing, Euclidean, Concat, GCN, and Kernel

DrugBank (https://go.drugbank.com/), containing all small-molecule drugs and their approved targets (data collected on 2025/04/07), was used as a dataset for DTI prediction. Entries that lacked SMILES or target information were excluded. The number of positive samples, representing DTI, was counted, and negative examples were randomly sampled at a 10:1 ratio to the positives. The dataset was divided into training, validation, and test sets in an 8:1:1 ratio. We constructed the SYSTCM-TNet, comprising 20,577 positive and 204,667 negative samples. On average, each target was associated with 4.48 drugs, while each drug interacted with 2.47 targets (Fig. 2A).

Molecular embeddings were obtained using the ChemBERTa-zinc-base-v1 pretrained model, which is a transformer-based language model trained on millions of chemical compounds to capture semantic and structural features of molecules. Each molecule was represented by its SMILES and encoded into a 768-dimensional embedding vector. Additionally, Extended-Connectivity Fingerprints (ECFPs) were computed using RDKit for each small molecule, with a radius of 2.0 and 2048 bits.

Targets were subsequently mapped to their corresponding protein sequences. Protein embeddings were generated using the ESM-2 pretrained model, a state-of-the-art protein language model trained on large-scale sequence data to learn biologically meaningful protein representations [16]. Each protein sequence was input into the model and encoded into a 2560-dimensional embedding vector. Gene Ontology (GO) features retrieved from UniProt were encoded as 12,899-dimensional binary vectors and reduced to 256 dimensions using Principal Component Analysis.

The molecular embedding and ECFP fingerprint were concatenated to form the final drug representation, while the protein embedding and GO feature vector were concatenated to construct the target representation.

### SYSTCM-TNet architecture

The KAN layer, designed based on Kolmogorov–Arnold representation theory, serves as an alternative to conventional MLPs [17] (Fig. 2B). Unlike standard MLPs that rely on deep stacking of fully connected layers, KANs achieve nonlinearity through the composition of learnable univariate transformations and additive interactions. This design enables expressive representation with better generalization and improved training stability, especially when handling high-dimensional biological data. According to the Kolmogorov-Arnold representation theorem, any multivariate continuous function can be expressed as a superposition of univariate continuous functions. Formally:

1 $$cf(x_{1} ,x_{2} , \ldots ,x_{n} ) = \Sigma _{{q = 0}}^{{2n}} \,\Phi _{q} \left( {\Sigma _{{p = 1}}^{n} \,\psi _{{q,p}} \left( {x_{p} } \right)} \right)$$

where ${\phi}_{q}$ and ${\psi}_{q,p}$ are learnable univariate continuous functions. The KAN layer leverages this principle by constructing networks that directly approximate such functional forms, thereby improving the model’s capacity to learn complex relationships.

Specifically, the drug and target vectors are independently processed by two parallel KAN branches. Each KAN branch treats every input feature as the input to a learnable univariate function $\psi \left({x}_{i}\right)$, which captures nonlinear patterns specific to each dimension. The outputs of these transformations are then additively combined and passed through a second-stage univariate function ϕ, yielding drug and protein representations that capture higher-order interactions.

During mini-batch construction, we adopt a drug-anchored hard negative mining strategy to emphasize confounding, near-positive negatives. For a drug ${d}_{i}$ with positive targets $P\left(i\right)$ and negatives $N\left(i\right)$, we randomly draw one positive ${t}^{+}\in \mathcal{P}(i)$. Given a current scoring model $s\left(d,t\right)$ (the forward pass is run in evaluation mode without gradients), we rank candidate negatives $t\in N\left(i\right)$ by their similarity to ${d}_{i}$ and select the top-K hardest:

2 $${\mathcal{H}}_{\mathcal{K}}\left(i\right)=Top{K}_{t\in \mathcal{N}\left(i\right)}s\left({d}_{i},t\right)$$

To model semantic alignment between drug and protein embeddings, we employed a multi-head attention mechanism. The KAN-encoded drug embedding $D\in {R}^{h}$ serves as the query, while the protein embedding ${\boldsymbol{T}}\in {R}^{h}$ acts as both key and value.

The vectors are first linearly transformed:

3 $$Q=D{W}_{Q},K=T{W}_{K},V=T{W}_{V}$$

where ${W}_{Q},{W}_{K},{W}_{V}\in {\mathbb{R}}^{h\times h}$ are learnable projection matrices. Scaled dot-product attention was then computed as:

4 $$Attention\left(Q,K,V\right)=softmax\left(\frac{Q{K}^{{\top}}}{\sqrt{h}}\right)V$$

The attention output $A\in {R}^{h}$ was concatenated with the original drug embedding:

5 $$C=\left[D;A\right]\in {R}^{2h}$$

and passed through a fusion layer:

6 $$Z=ReLU\left({W}_{f}C+{b}_{f}\right),{W}_{f}\in {R}^{h\times 2h}$$

Both the fused drug vector and the protein vector were L2-normalized:

7 $$\widehat{Z}=\frac{Z}{||Z|{|}_{2}},\widehat{T}=\frac{T}{||T{||}_{2}}$$

and their cosine similarity was computed as the final interaction score:

8 $$s=\widehat{{Z}^{{\top}}}\widehat{T}$$

which quantifies the alignment and compatibility between drug and protein representations.

To optimize drug–target interaction matching, we employed Circle Loss, a ranking-based objective designed to adaptively balance positive and negative sample separation. Let ${s}^{+}$ denote the similarity score for an interacting drug–target pair and ${s}_{i}^{-}$ the scores for non-interacting pairs. The loss is defined as:

9 $$\mathcal{L}=\mathrm{log}\left(1+{\sum}_{i=1}^{N}\mathrm{exp}\left[\gamma \left({s}_{i}^{-}-m\right)\right]\right)+\mathrm{log}\left(1+\mathrm{exp}\left[-\gamma \left({s}^{+}-\left(1-m\right)\right)\right]\right)$$

where m is the margin hyperparameter, and γ is a scale factor. This formulation encourages positive scores to exceed 1-m while penalizing negative scores that surpass m, thereby providing flexible separation of difficult cases and improving model robustness. In our model, the margin m was set to 0.25 and the scaling factor γ was set to 64. Model training was optimized using an early stopping strategy with a patience parameter of 10.

### Overall performance comparison

We compared the performance of SYSTCM-TNet with multiple baseline methods, which include Cosine [18], Concat [19], Euclidean [20], Kernel [21], FeatureCrossing [22], and GCN algorithms [23] (Fig. 2C). These baselines represent widely adopted model architectures and provide meaningful performance references. The source code for SYSTCM-TNet and all baseline implementations is publicly available at https://github.com/SYSTCM/FuTCM-PDD.

To comprehensively evaluate the ranking performance of SYSTCM-TNet and baseline models in predicting drug–target interactions, we adopted several widely used metrics from recommendation systems, including Recall@K, Normalized Discounted Cumulative Gain (NDCG@K), Mean Average Precision (MAP), and Mean Reciprocal Rank (MRR). Let D denote the test set, and ${T}_{d}^{+}$ be the set of true targets associated with drug d∈D.

10 $$Recall@K=\frac{1}{\left|\mathcal{D}\right|}{\sum}_{d\in \mathcal{D}}\frac{\left|Top-K\left(d\right)\cap {T}_{d}^{+}\right|}{\left|{T}_{d}^{+}\right|},K\in \{\mathrm{1,3},\mathrm{5,10}\}$$

$Top-K\left(d\right)$ denotes the top K predicted targets for drug. Higher Recall@K indicates that the model can retrieve a larger portion of known binding targets among the most promising candidates.

11 $$NDCG@K\left(d\right)=\frac{DCG@K\left(d\right)}{IDCG@K\left(d\right)}, DCG@K\left(d\right)={\sum}_{i=1}^{K}\frac{{rel}_{d,i}}{{\mathrm{log}}_{2}\left(i+1\right)}$$

Here, ${rel}_{d,i}$∈ {0,1} indicates whether the target ranked at position i is a true interaction for drug d. $IDCG@K\left(d\right)$ is the ideal DCG, where all true targets are ranked at the top.

12 $$MAP=\frac{1}{\left|\mathcal{D}\right|}{\sum}_{d\in \mathcal{D}}\frac{1}{\left|{T}_{d}^{+}\right|}{\sum}_{k=1}^{n}{P}_{d}\left(k\right)\cdot {rel}_{d,k}$$

where ${P}_{d}\left(k\right)$ is the precision at cutoff k, and ${rel}_{d,k}$∈ {0,1} denotes whether the target at rank k is relevant, and n is the total number of ranked targets. MAP captures the overall ranking quality across all positives.

13 $$MRR=\frac{1}{\left|\mathcal{D}\right|}{\sum}_{d\in \mathcal{D}}\frac{1}{{rank}_{d}}$$

where ${rank}_{d}$ is the position of the first correctly predicted target for drug d.

We used an early-stopping criterion based on a weighted score, computed as *Recall@10* + *3.0* × *MAP* + *2.0* × *MRR*. If this metric failed to improve over 10 successive epochs, training was stopped.

### Gradient-based model interpretability analysis

To assess whether SYSTCM-TNet captured biologically meaningful molecular structural features and protein functional features, we performed Gradient × Input attribution analysis using the trained model without further parameter updating. For each positive drug–target pair in the validation set ${\mathcal{V}}^{+}$, the model prediction score was denoted as ${f}_{\theta }\left({d}_{i},{t}_{i}\right)$. For the *j-th* ECFP bi ${e}_{ij}$, its attribution score was calculated as:

14 $${A}_{ij}^{ECFP}={e}_{ij}\frac{\partial {f}_{\theta }\left({d}_{i},{t}_{i}\right)}{\partial {e}_{ij}}$$

The overall importance of this ECFP bit was then defined as the mean absolute attribution across all validation positive samples:

15 $$I_{j}^{ECFP} = \frac{1}{{\left| {\nu^{ + } } \right|}}\Sigma_{{i \in \nu^{ + } }} \left| {A_{ij}^{ECFP} } \right|$$

Similarly, for the *k-th* GO-PC feature ${g}_{ik}$, its attribution score was calculated as:

16 $${A}_{ik}^{GO}={g}_{ik}\frac{\partial {f}_{\theta }\left({d}_{i},{t}_{i}\right)}{\partial {g}_{ik}}$$

and its overall importance was defined as:

17 $$I_{k}^{{GO}} = \frac{1}{{\left| {v^{ + } } \right|}}\Sigma _{{i \in v^{ + } }} \left| {A_{{ik}}^{{GO}} } \right|$$

The top 50 ranked ECFP bits and the top 50 ranked GO-PC features were selected as high-contribution features. To validate their functional relevance, these selected features were masked to zero in the independent test set, and model performance was re-evaluated using Recall@10, MAP@10, MRR@10, and NDCG@10. As a negative control, the same number of ECFP bits or GO-PC features was randomly masked five times.

Because GO-PC features were generated from original GO annotations by PCA, the PCA loading matrix L was used to interpret high-contribution GO-PC features. For each selected GO-PC feature, representative GO terms were ranked according to the absolute loading values $\mid {L}_{kq}\mid$. In addition, the back-projected importance of the q-th GO term was calculated as:

18 $$B_{q} = \sum\nolimits_{{k \in S_{{50}}^{{GO}} }} {I_{k}^{{GO}} } \left| {L_{{kq}} } \right|$$

where ${S}_{50}^{GO}$ denotes the set of top 50 GO-PC features. This analysis was used to identify original GO annotations associated with high-contribution protein functional features.

To further examine whether SYSTCM-TNet learned nonlinear functional-response patterns, partial dependence plots (PDPs) were generated for the top-ranked GO-PC features. For a selected GO-PC feature ${g}_{ik}$, its value was replaced by a series of values v observed in the validation set while all other input features were kept unchanged. The average prediction score was then calculated as:

19 $$PDP_{k} (v) = \frac{1}{{\left| {v^{ + } } \right|}}\sum\nolimits_{{i \in v^{ + } }} {f_{\theta } \left( {d_{i} ,t_{i} ;g_{{ik}} = v} \right)}$$

where ${f}_{\theta }\left({d}_{i},{t}_{i};{g}_{ik}=v\right)$ denotes the model prediction score after replacing only the *k-th* GO-PC feature with v. Each GO-PC feature in the PDP analysis was annotated using the representative GO terms with the largest absolute PCA loadings.

### Ablation experiments

To assess the contribution of individual architectural components within SYSTCM-TNet, we performed a series of ablation experiments. These ablations covered three major aspects: feature embedding dimensions, model architecture, and optimization strategies. For each ablation, a single module was removed or replaced while all other settings were kept identical, and the resulting models were evaluated on the same dataset using the same metrics. The ablation configurations were as follows:

*SYSTCM-TNet* the complete model incorporating all designed components.

*TNet-NoECFP, TNet-NoGO, and TNet-NoEG* variants in which the ECFP embedding, GO term embedding, or both were removed, used to assess the individual and combined contributions of chemical structure and functional annotations.

*TNet-MLP* a variant where the KAN module was replaced with an MLP of equivalent parameter size, designed to examine the necessity of domain-aware encoding provided by KAN.

*TNet-NoAttn* a variant in which the multi-head attention mechanism was removed, used to evaluate its role in capturing inter-feature dependencies.

### Molecular docking

The three-dimensional structures of protein targets were primarily obtained from the RCSB Protein Data Bank (https://www.rcsb.org/). For proteins without available crystal structures, high-confidence models were retrieved from AlphaFold (https://alphafold.com/). Protein structures were preprocessed using Discovery Studio 4.0 (DS), including removal of water molecules, correction of missing loops, and addition of hydrogen atoms.

Small-molecule ligands were obtained from the PubChem database and subjected to energy minimization to optimize their conformations. Binding pockets were defined based on the co-crystallized ligand in cases where experimental receptor-ligand complexes were available. For AlphaFold-predicted receptors lacking bound ligands, potential binding sites were predicted using cavity detection algorithms, and the top-ranked predicted site was selected as the active site.

Molecular docking was performed using the CDOCKER module in DS. Docking results were evaluated based on -CDOCKER interaction energy scores and receptor-ligand binding interactions. Visualization and further analysis of binding poses were conducted using PyMOL.

### Data collection and processing of CMM

We collected all CMMs with their recorded efficacies and properties from the *Chinese Pharmacopoeia*. The efficacies were split into individual components, for example, *activating blood flow and removing blood stasis* was separated into *activating blood flow* and *removing blood stasis*. In the next step, based on *Traditional Chinese Pharmacy*, *Efficacy of Chinese Materia Medica*, we standardized all efficacies into 18 distinct categories (Table S4). For the five flavors, we included the traditional categories: *bitter, sour, pungent, salty,* and *sweet*. Non-traditional flavors, such as “*astringent*”, were excluded. Regarding the meridians, the *Sanjiao* and *Pericardium* meridians were excluded due to an insufficient number of samples, with only 4 and 7 entries respectively.

The 40 pharmacological effects were standardized from common therapeutic effects in modern medicine, following the criteria established in our previous work [24]. For each pharmacological effect, we searched CNKI using the combination of the effect term and the name of each CMM as keywords, restricting the search scope to titles, keywords, and abstracts. The number of retrieved publications was recorded for each CMM–pharmacological effect pair.

For a given pharmacological effect e and CMM t, if the number of associated publications ${Count}_{t,e}$ exceeded 10, the pair was considered valid and encoded as 1; otherwise, it was encoded as 0:

20 $${R}_{t,e}=\left\{\begin{array}{c}1, if {Count}_{t,e}\ge 10\\ 0, otherwise\end{array}\right.$$

By this procedure, we obtained a binary relationship matrix representing pharmacological effect–CMM associations. Using pharmacological effects as input features and either efficacies or properties as labels, we constructed multiple datasets for supervised binary classification tasks. In addition, all quality-control compounds of each CMM were retrieved from the *Chinese Pharmacopoeia* to generate pharmacological effect–compound pairs, which were incorporated as constraint data for model development.

### Model construction of efficacies and properties

We established a lightweight AutoML pipeline using the OPTUNA framework [25, 26], integrating feature selection, resampling, and classification algorithms into a total of 168 algorithmic combinations. Feature selection methods included Mutual Information Coefficient (FSMIC), Chi-Square (Chi2), Random Forest (RF), and Support Vector Classification-Recursive Feature Elimination (SVC-RFE). Resampling strategies encompassed SMOTE, SVM-SMOTE, Borderline-SMOTE, ClusterCentroid, Neighborhood Cleaning Rule (NM), and the hybrid SMOTE-ENN. For classification, we considered K-Nearest Neighbors (KNN), Support Vector Machine (SVM), Random Forest (RF), Multi-Layer Perceptron (MLP), Logistic Regression (LR), XGBoost, and AdaBoost.

For each algorithm, key hyperparameters were tuned within predefined ranges using OPTUNA with five-fold cross-validation (5-CV). The complete search space for all algorithms is summarized in Table 1. Finally, we quantified and visualized the occurrence frequencies of individual algorithms and their combinations to highlight dominant optimization patterns. We further introduced a proportion-based constraint regularization term to encourage distributional consistency between Chinese Materia Medica-level labels and quality-control compound-level labels [27, 28].

**Table 1:** Hyperparameter configurations for each algorithm

Category	Algorithm	Hyperparameter	Range/Options
Feature Selection	FSMIC	Number of selected features	5–30
	Chi2	Number of selected features	5–30
	RF	Feature importance threshold	0.01–0.02
	SVC-RFE	Number of selected features	5–30
Resampling	SMOTE	Class distribution ratio	1:1
	SVM-SMOTE		
	Borderline-SMOTE		
	ClusterCentroids		
	NearMiss		
	SMOTE-ENN		
Classification	KNN	Number of neighbors (k)	2–32, step = 2
		Weighting method	{‘uniform’, ‘distance’}
	SVM	Kernel type	{‘inear’, ‘rbf’, ‘sigmoid’}
		Gamma	1e-5–1e5
		Regularization parameter (C)	1e-10–1e-2
	RF	Maximum depth	2–32
		Number of estimators	10–500
	LR	Regularization strength (C)	1e-2–1e2
		Penalty type	{‘l1’, ‘l2’}
		Solver	{‘liblinear’, ‘saga’}
	XGBoost	Number of estimators	10–500
		Learning rate (eta)	0.01–0.3
		Booster type	{‘gbtree’, ‘gblinear’, ‘dart’}
		Objective function	{‘logistic’, ‘hinge’}
	AdaBoost	Number of estimators	10–500
		Learning rate	0.01–0.3
		Boosting algorithm	{‘SAMME.R’, ‘SAMME’}
	MLP	Solver	{‘adam’, ‘lbfgs’, ‘sgd’}
		Activation function	{‘relu’, ‘tanh’, ‘logistic’}
		Learning rate strategy	{‘invscaling’, ‘constant’, ‘adaptive’}
		Number of hidden layers	1–5
		Neurons per hidden layer	16–256

Because reliable ground-truth annotations of TCM efficacies and properties are currently unavailable at the individual-compound level, the primary supervised-learning task was established exclusively at the Chinese Materia Medica level [29]. After each candidate model was trained using the Chinese Materia Medica-level samples, it was applied to the quality-control compounds to generate predicted efficacy or property labels. The class proportions of the quality-control compounds were then automatically calculated from these model-predicted labels. To avoid selecting models that produced a compound-level class distribution markedly inconsistent with the empirical label distribution of the Chinese Materia Medica training set, we introduced a proportion-based model-selection objective into the OPTUNA framework:

21 $${\mathcal{L}}={\sum}_{k=1}^{K}\left|{P}_{k}^{\left(H\right)}-{P}_{k}^{\left(Q\right)}\right|$$

where ${P}_{k}^{\left(H\right)}$ and ${P}_{{\boldsymbol{k}}}^{\left(Q\right)}$ denote the proportion of positive samples in category k among CMMs and quality control compounds, respectively.

At the end, we trained the model using fivefold cross-validation and evaluated its performance with Matthews Correlation Coefficient (MCC), Area Under the ROC Curve (AUC), F1-score, precision (PRE), recall (REC), and accuracy (ACC). For the cold–hot–neutral property classification, which is a three-class task, metrics were calculated using macro-averaging, which computes the unweighted mean of the metric across all classes. For all other binary classification tasks, standard binary evaluation methods were applied.

### JACCARD analysis and association rule mining

To quantify the associations among pharmacological effects, CMM properties, and efficacies, we calculated the Jaccard similarity between their corresponding label sets. For instance, the similarity between efficacies (E) and properties (P) was defined as:

22 $$Jaccard\left(E,P\right)=\frac{\left|{\mathcal{Y}}^{\left({\boldsymbol{E}}\right)}\cap {\mathcal{Y}}^{\left({\boldsymbol{P}}\right)}\right|}{\left|{\mathcal{Y}}^{\left({\boldsymbol{E}}\right)}\cup {\mathcal{Y}}^{\left({\boldsymbol{P}}\right)}\right|}$$

where ${\mathcal{Y}}^{\left({\boldsymbol{E}}\right)}$ and ${\mathcal{Y}}^{\left({\boldsymbol{P}}\right)}$ denote the sets of assigned categories for a given CMM under efficacies and properties respectively. Jaccard similarities were computed for all pairwise combinations among these three category sets, and the results were visualized as a heatmap.

To characterize how AutoML combined different learning algorithms, we performed association rule mining on the algorithms selected across all AutoML runs. For each run, the algorithms included in the final model were encoded as a single transaction, with individual algorithms treated as items. Association rules of the form A ⇒ B were extracted to capture frequently co-occurring algorithm pairs or sets. For each rule, we calculated support and confidence.

### AI-driven prediction and integration of multi-level CMM information

CMMs, compounds, and targets were retrieved from TCMBank [30], HIT 2.0 [12], and peer-reviewed literature. Canonical compound structures were obtained from PubChem in SDF format, and duplicate molecules were removed using the *Prepare Ligand* module of DS. Targets supported by literature evidence were retained, and all compounds were subsequently subjected to target prediction using the SYSTCM-TNet model. Targets were deemed positive if their scores exceeded μ + 3σ, where μ and σ denote the mean and standard deviation across all scores.. Besides, all compounds were predicted pharmacology effects, efficacies and properties by the model [24]. Finally, CMM herbs, compounds, and predicted targets were each assigned a unique identifier for downstream integration.

### Construction of the property/efficacy network and randomization analysis

For each predefined property or efficacy, we first collected all CMMs and compounds associated with the attribute. We then established CMM–compound and compound–target associations. Functional enrichment of these targets was performed with adjusted *P*-value < 0.05, and the significant pathways were included as pathway nodes. Pharmacological effects annotated for each CMM or compound were integrated into the network by adding CMM–Pharmacological Effect and compound– pharmacological effect edges. Finally, a total of 36 property/efficacy-specific networks were constructed, each comprising CMMs, compounds, targets, pathways, and pharmacological effects.

To demonstrate the biological and statistical significance of the property/efficacy-specific networks, we employed two distinct randomization strategies to construct degree-preserving null models. (1) In the edge-rewired randomization, pairs of edges were iteratively swapped while prohibiting self-loops and multi-edges, thereby strictly preserving each node’s degree. (2) In the heterogeneous network randomization, each edge was reassigned between two nodes of the same attribute class, preserving both individual node degrees and the global class-to-class edge counts. Each ensemble consisted of 10, 000 randomized realizations, from which the means (µ) and standard deviations (σ) were used to generate null distributions. Statistical significance was assessed by computing z-scores ($z=\left({x}_{obs}-\mu /\upsigma \right)$), with deviations considered significant at two-tailed *P* < 0.05. For the edge-rewired ensemble, significance was evaluated using average shortest-path length and average clustering coefficient. For the heterogeneous ensemble, additional metrics—network diameter, average degree centrality, and degree heterogeneity—were also assessed. Formal definitions of all metrics are provided in Table 2.

**Table 2:** Topological metrics and analytical definitions

Symbol	Formula	Interpretation
Average shortest path length	$L = \frac{1}{{N\left( {N - 1} \right)}}\sum\limits_{i \ne 1} {d\left( {i,j} \right)}$	Average length of the shortest route between any two nodes
Average clustering coefficient	$C = \frac{1}{N}\sum\limits_{(i = 1)}^{N} {\frac{{2e_{i} }}{{k_{i} (k_{i} - 1)}}}$	Mean clustering level of nodes across the entire network
Network diameter D	$D=\underset{i,j}{\mathrm{max}}d\left(i,j\right)$	Longest of all shortest routes found between any pair of nodes
Average degree centrality k	$\overline{k} = \frac{1}{N}\sum\limits_{(i = 1)}^{N} {k_{i} }$	Average number of edges attached to a node
Degree heterogeneity H	$H=\frac{\langle {k}^{2}\rangle }{{\langle k\rangle }^{2}}$	Degree to which node connections vary within the network

N, total number of nodes in the network, $d\left(i,j\right)$, shortest-path length between nodes, i and *j*; ${k}_{i}$ degree of node i, ${e}_{i}$ number of edges among the ${k}_{i}$ neighbours of node i, k and $\langle {k}^{2}\rangle$ denote the first and second moments of the degree distribution, respectively

### Quantitative scoring framework for CMMs–compounds–targets–pathways associations with properties/efficacies

For each property/efficacy-specific network, we designed a three-layer scoring framework to quantify node importance.

Heterogeneous network centrality (H) was defined as a weighted combination of three classical topological indices:

23 $$H=0.3\cdot Deg+0.4\cdot Bet+0.3\cdot PR$$

where Deg, Bet, and PR denote degree centrality, betweenness centrality, and PageRank, respectively.

Information flow importance (I) was calculated using an iterative diffusion model. For each node type, all nodes of that type were treated as sources and initialized with equal flow. At each iteration, a fraction (1 − α) of the flow was retained by the sources, while the remaining fraction α was equally distributed to their neighbors:

24 $${F}^{\left(t+1\right)}\left(v\right)=\left(1-\upalpha \right)\cdot S\left(v\right)+\upalpha {\sum}_{u\in \mathcal{N}\left(v\right)}\frac{{F}^{\left(t\right)}\left(u\right)}{\left|\mathcal{N}\left(u\right)\right|}$$

where $S\left(v\right)$ is the source initialization, $\mathcal{N}\left(u\right)$ is the neighbor set of node u, and α=0.85. After 50 iterations, flows were aggregated across node types and normalized. Finally, node-specific type weights ${w}_{type\left(v\right)}$ were applied to yield the importance score:

25 $$I\left(v\right)={w}_{type\left(v\right)}\cdot \frac{F\left(v\right)}{\underset{u}{\mathrm{max}}F\left(u\right)}$$

To assess the robustness of embeddings against node removal, we embedded the original network using Node2Vec and then iteratively deleted node v. The perturbation sensitivity (P) was quantified as the average Euclidean distance between embeddings of the remaining nodes before and after removal:

26 $$P\left(v\right)=\frac{1}{\left|U\right|}{\sum}_{u\in U}|{z}_{u}-{z}_{u}{\prime}{|}_{2}$$

where $U=V\left(G\right)\cap V\left({G}_{-v}\right)$ is the set of common nodes between the original and perturbed graphs, and ${z}_{u}, {z}_{u}{\prime}$ denote the embeddings of node u before and after perturbation.

Because perturbation analysis is computationally expensive, only the top 10% of nodes within each node type, ranked by the meaning of H and I, were subjected to perturbation evaluation. Accordingly, the final node importance score was defined as a piecewise function:

27 $$Score\left(v\right)=\left\{\begin{array}{c}0.3H+0.3I+0.4P , v\in Top10\% \\ 0.5H+0.5I, otherwise\end{array}\right.$$

Finally, for nodes appearing across multiple networks, the scores were normalized by applying a softmax transformation:

28 $$FinalScore\left(v\right)=softmax\left(Score\left(v\right)\right)$$

### Network pharmacology

Compounds and their corresponding targets were collected from the previous dataset, while disease-related targets were sourced from GeneCards (https://www.genecards.org/) and OMIM (https://www.omim.org/). A Venn analysis was performed to identify the intersection between the CMM targets and disease targets. The resulting network was visualized and analyzed using Cytoscape. For the efficacy network, only the compounds associated with specific efficacy were included, along with their corresponding targets. The analysis for this network followed the same procedure as for the conventional network. Degree rank was used to identify the active compounds of CMM.

### Materials

The HepG2 cells were purchased from Servicebio (Wuhan, China). NRR was obtained from Tongrentang Medicine Corporation Ltd. (Beijing, China). 5-geranoxy-7-methoxycoumarin (GMOC), alloisoimperatorin (AIP), Bornyl Acetate (BA) were purchased from PUSH BIO-TECHNOLOGY (Chengdu, China)**.** DMEM was purchased from VivaCell (Shanghai, China), and the CCK-8 assay kit was obtained from LABLEAD (Beijing, China). Fetal bovine serum and penicillin–streptomycin were purchased from Aoqing Biotechnology (Beijing, China). Palmitic acid and oleic acid were purchased from Kunchuang Biotechnology (Xi'an, China).

### Preparation for NRR extraction

NRR (10 g) was powdered and extracted with 8 volumes of 75% ethanol (30 min maceration, 2 h reflux), filtered, and the marc was re-extracted once with 8 volumes of water. Combined filtrates were lyophilized to obtain the NRR extract (NRRE), which was dissolved in DMSO for cell assays.

### Cell culture and viability assay

HepG2 cells were maintained in DMEM supplemented with 10% FBS and 1% penicillin–streptomycin at 37 °C in 5% CO₂. Cell viability was assessed using the CCK-8 assay by measuring absorbance at 450 nm. Cell viability was calculated as follows: The cell survival rates are calculated as [(ODdrug-ODblank)/ (ODcontrol-ODblank)] × 100%.

### Oil Red O staining

Cells were seeded in 96-well plates at 3 × 10^4^ cells/well and, after 24 h, a lipid-overload model was induced using 500 μM oleic acid and 250 μM palmitic acid for 24 h. Thereafter, NRRE and the indicated active compounds were administered at their maximum non-cytotoxic concentrations for an additional 24 h. Cells were fixed with 4% paraformaldehyde for 20 min and washed three times with PBS. Following a 60% isopropanol rinse, cells were stained with Oil Red O working solution in the dark for 15 min, washed with PBS, and imaged by light microscopy. Lipid accumulation was semi-quantified from micrographs using ImageJ.

## Results

### Overall performance of SYSTCM-TNet

**Fig. 3:**
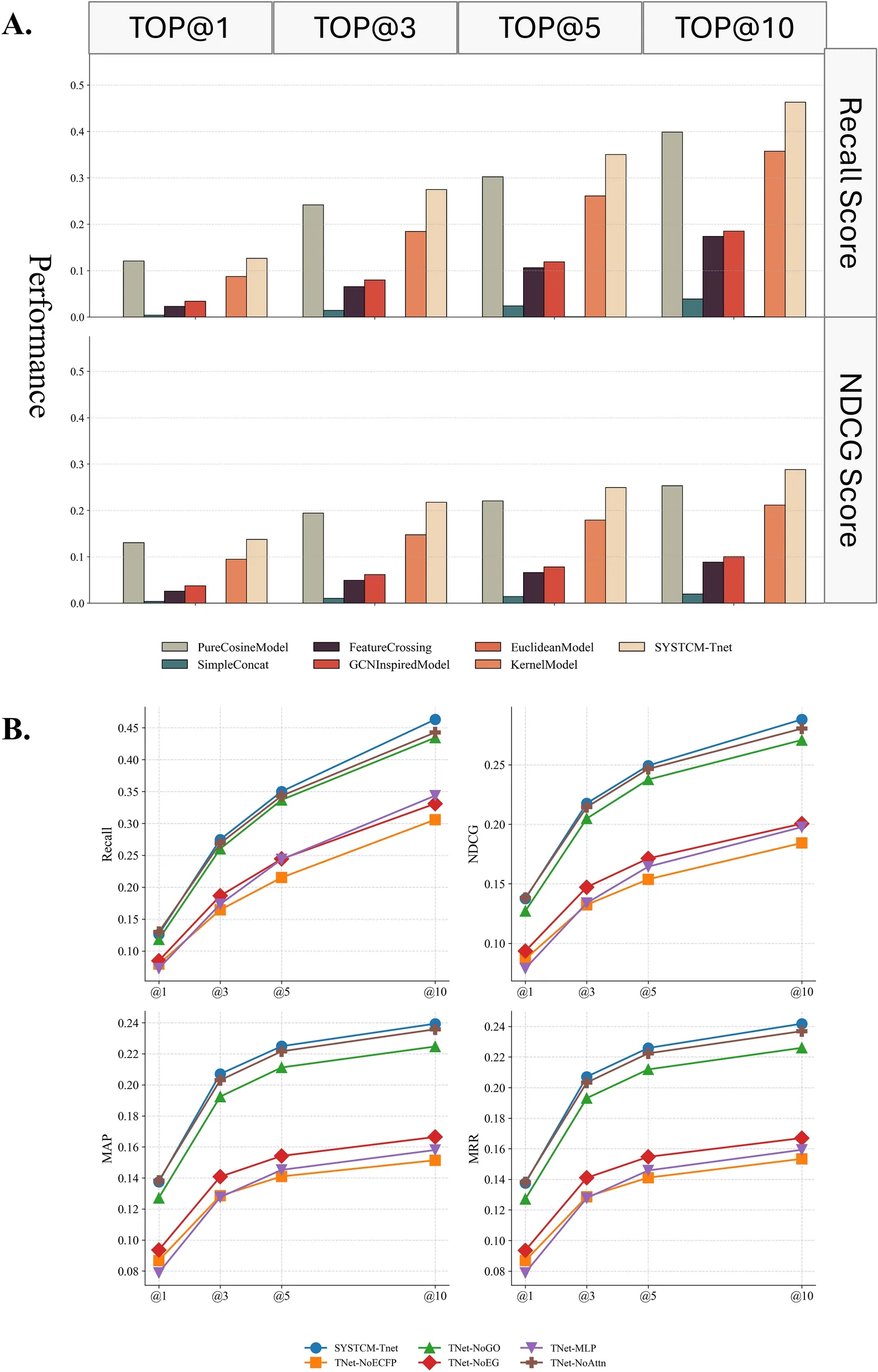
Overall performance comparison and ablation analysis of SYSTCM-TNet. **A** Performance comparison of baseline model **B** Ablation study on key modules of SYSTCM-TNet

SYSTCM-TNet was developed using a KAN architecture and optimized with Circle Loss. The model was trained and evaluated based on the DrugBank. The performance of SYSTCM-TNet was compared with six baseline models, including FM, GCN, kernel-based methods, and others. As shown in Fig. 3A, SYSTCM-TNet consistently outperformed six baseline methods across the evaluated ranking metrics. Among the baseline models, PureCosine achieved the highest performance, with a Recall@10 of 0.3987, NDCG@10 of 0.2530, MAP@10 of 0.2016, and MRR@10 of 0.2156. In contrast, SYSTCM-TNet achieved the best results with a Recall@10 of 0.4631, NDCG@10 of 0.2880, MAP@10 of 0.2394, and MRR@10 of 0.2418. These results represent relative improvements of + 16.14% in Recall, + 13.83% in NDCG, + 15.78% in MAP, and + 12.15% in MRR over the best-performing baseline [31], indicating that SYSTCM-TNet not only improves the retrieval of relevant targets but also enhances their ranking positions within the top predictions [32]. Compound cold-start validation and ChEMBL external validation indicated that SYSTCM-TNet retained a certain degree of generalization ability for unseen compounds within its defined chemical applicability domain (Table S2 and Table S3).

To assess the contribution of individual modules and input features in SYSTCM-TNet, we performed a series of ablation studies (Fig. 3B). The complete model consistently outperformed all ablated variants. Notably, the removal of ECFP molecular fingerprints and KAN layers resulted in the maximum performance declines—exceeding 30% across all metrics—underscoring their critical roles in drug representation and knowledge-guided interaction modeling. Excluding GO annotation features led to moderate performance drops (6–10%), while removing the attention mechanism yielded relatively minor but consistent degradations (1–4%), indicating its effectiveness in capturing fine-grained feature interactions. Interestingly, the simultaneous removal of both GO annotations and ECFP features did not lead to a larger decline than removing ECFP alone. Specifically, dual ablation resulted in a 28.53% drop in Recall@10, compared to 33.92% when only ECFP was excluded. This suggests that GO and ECFP may contain partially overlapping or redundant information, and that the model may partially compensate for their joint absence by leveraging alternative features or adjusting internal representations. Overall, these results highlight the synergistic effects of multi-source biological features and structured attention mechanisms in enhancing the robustness and predictive performance of SYSTCM-TNet.

### SYSTCM-TNet captures interpretable molecular structural and protein functional features

**Fig. 4:**
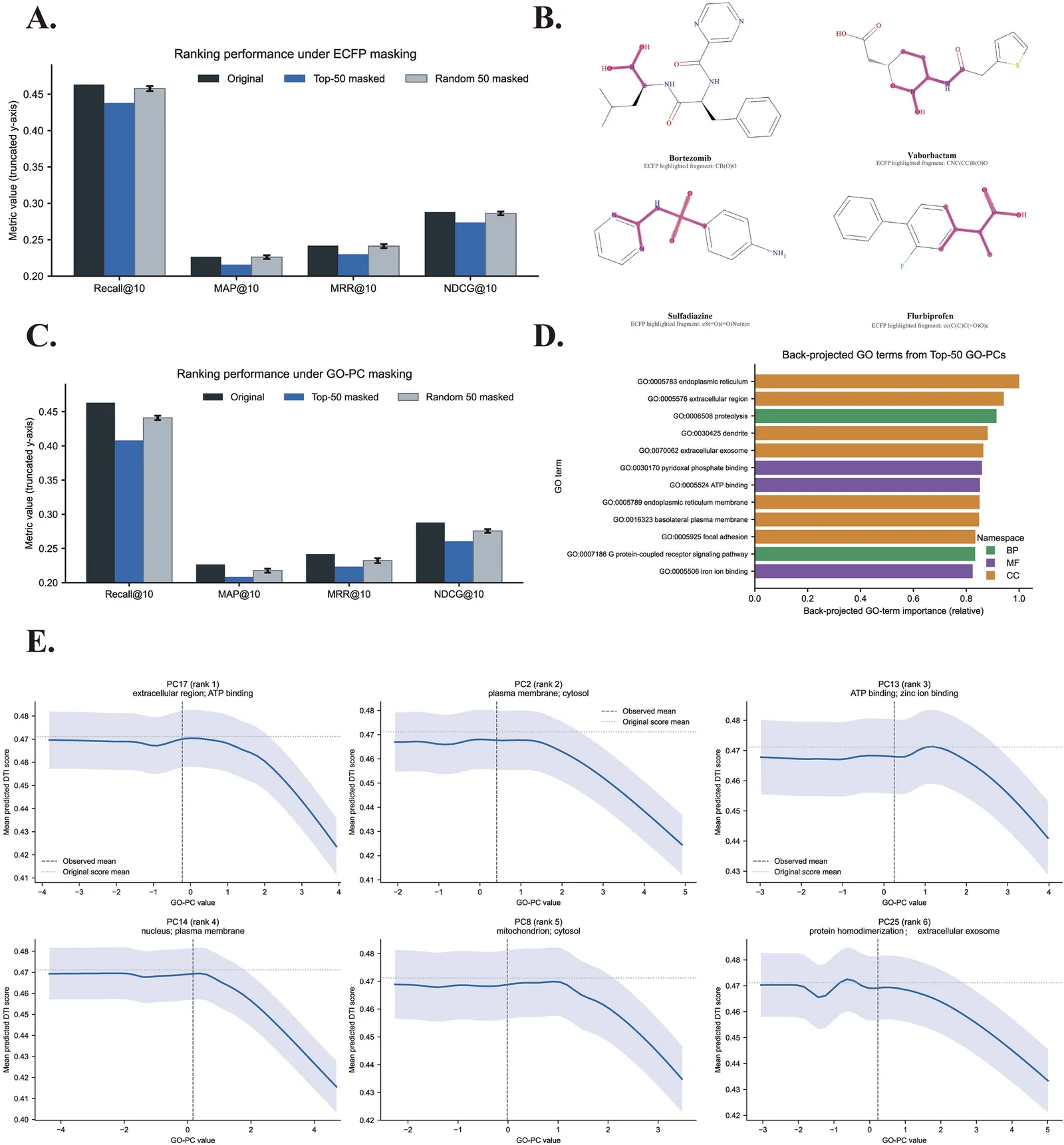
Interpretability analysis of SYSTCM-TNet based on molecular and protein functional features. **A** Ranking performance after masking the top 50 attributed ECFP bits or randomly selected 50 ECFP bits. **B** Representative high-contribution ECFP fragments mapped to known medicinal chemistry substructures in DrugBank. **C** Ranking performance after masking the top 50 attributed GO-PC features or randomly selected 50 GO-PC features. **D** Top back-projected GO terms derived from the attribution scores of the top 50 GO-PC features. **E** Partial dependence plots of representative top-ranked GO-PC features, showing nonlinear relationships between GO-PC feature values and predicted DTI scores

To further evaluate whether SYSTCM-TNet captured key features with drug–chemical and protein-functional relevance, we calculated Gradient × Input attribution scores for ECFP bits and GO-PC features. After masking the top 50 attributed ECFP bits, model performance decreased from 0.4631 to 0.4379 for Recall@10, from 0.2880 to 0.2736 for NDCG@10, from 0.2394 to 0.2157 for MAP@10, and from 0.2418 to 0.2299 for MRR@10, corresponding to relative decreases of 5.44%, 5.00%, 9.90%, and 4.92%, respectively (Fig. 4A). In contrast, randomly masking 50 ECFP bits caused only minor performance changes, indicating that the ECFP features identified by attribution analysis made higher contributions to model prediction. Mapping the high-contribution ECFP bits to representative molecular fragments revealed several well-known medicinal chemistry substructures, including boronic acid, sulfonamide-like, and arylpropionic acid motifs, as exemplified by bortezomib, vaborbactam, sulfadiazine, and flurbiprofen (Fig. 4B and Table S5). For example, the boronic acid or cyclic boronate groups of bortezomib and vaborbactam participate directly in their interactions with the proteasome and β-lactamases, respectively [33, 34] ; sulfonamide antibacterials act as structural analogues of para-aminobenzoic acid at dihydropteroate synthase; and the 2-arylpropionic acid scaffold is a characteristic structural feature of flurbiprofen and related cyclooxygenase inhibitors [35, 36]. These fragments constitute characteristic structural elements of the corresponding drug classes and are associated with their established chemical or pharmacological properties.

On the protein-functional side, masking the top 50 attributed GO-PC features also led to a more pronounced performance decrease (Fig. 4C and Table S5). Masking the top 50 attributed GO-PC features changed Recall@10 from 0.4631 to 0.4079, NDCG@10 from 0.2880 to 0.2605, MAP@10 from 0.2394 to 0.2083, and MRR@10 from 0.2418 to 0.2232, corresponding to relative decreases of 11.92%, 9.55%, 12.99%, and 7.69%, respectively. In comparison, randomly masking 50 GO-PC features resulted in a smaller decline. These results indicate that the high-attribution GO-PC features contained important protein functional information used by the model for drug–target prediction.

Because GO-PC features were derived from PCA-transformed GO annotations, we back-projected the attribution scores of the top 50 GO-PC features to the original GO-term space. The enriched high-importance GO annotations encompassed protein localization, binding activity, proteolysis, signal transduction, ATP binding, focal adhesion, and G protein-coupled receptor signaling (Fig. 4D).

Finally, we generated partial dependence plots (PDPs) for the top-ranked GO-PC features to examine the nonlinear functional-response relationships learned by the KAN model (Fig. 4E). Several high-contribution GO-PC features, including PC17, PC2, PC13, PC14, PC8, and PC25, showed nonlinear response patterns. When the values of these GO-PC features exceeded certain ranges, the model prediction scores exhibited marked decreases or local fluctuations rather than simple linear changes. This indicates that SYSTCM-TNet not only identified important protein functional features, but also modeled complex nonlinear relationships between these features and DTI prediction scores. Overall, the ECFP masking, GO-PC masking, GO-term back-projection, and PDP analyses collectively support the interpretability of SYSTCM-TNet and suggest that the key features captured by the model are consistent with known medicinal chemistry structures and protein functional principles.

### Evaluating the reliability of SYSTCM-TNet target Predictions through baicalein

To evaluate the reliability of the target prediction results generated by SYSTCM-TNet, baicalein, which has been reported to possess anti-cancer [37, 38], anti-inflammatory [39], and anti-Alzheimer’s disease activities [40], was selected as a case for target predictions based on SYSTCM-TNet. We adopted a three-layer validation strategy combining cross-referencing with CMM-related databases, literature mining, and structure-based molecular docking of the predicted targets.

**Fig. 5:**
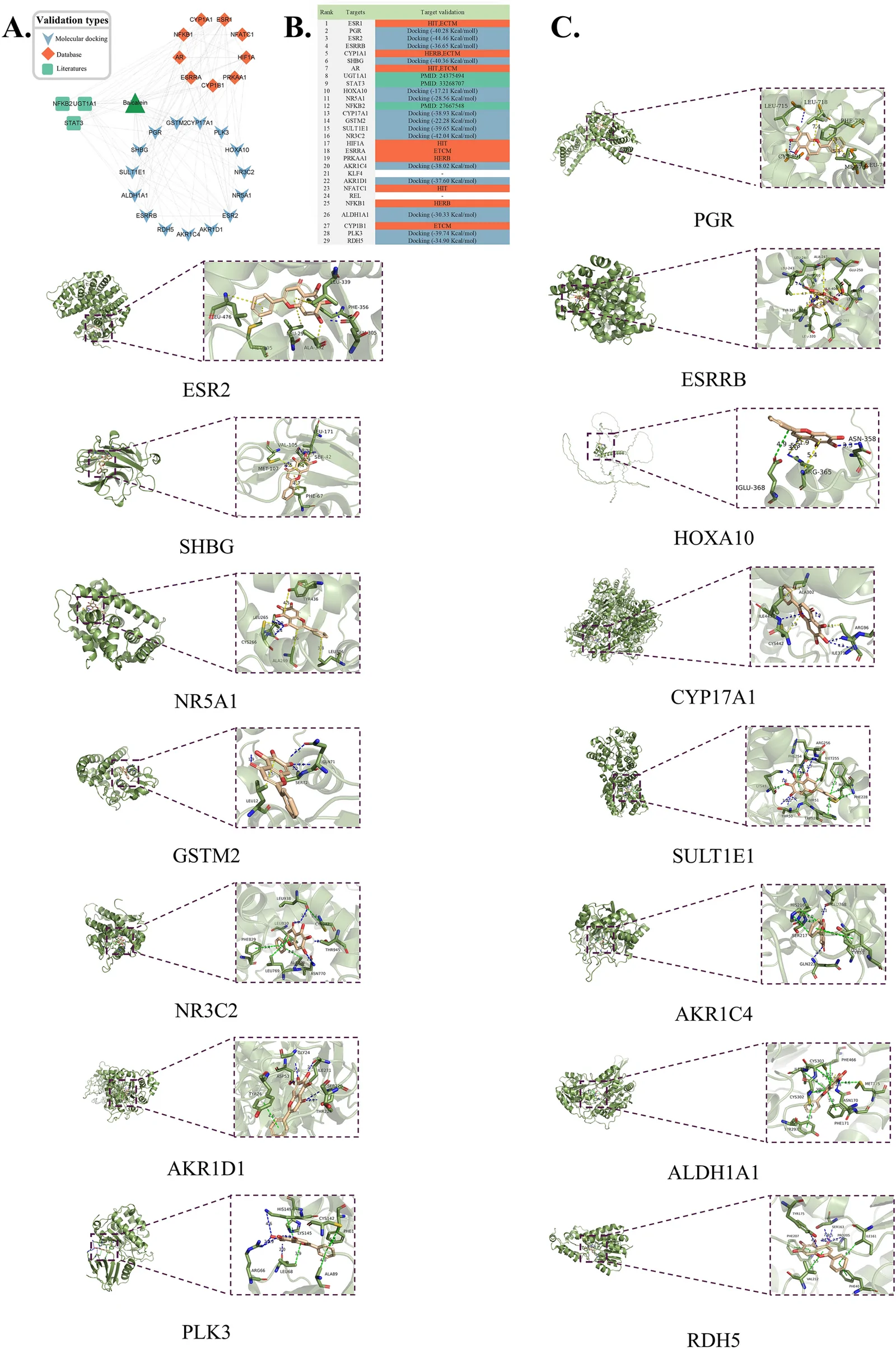
Validation of baicalein-target predictions by database, literature, and molecular docking. **A** Network of baicalein and its 29 predicted protein targets. Targets validated by database entries (green triangles), literature evidence (red diamonds), and molecular docking (blue circles) are marked accordingly. **B** Summary table of all 29 predicted targets, with validation type and source information. **C** Molecular docking results of baicalein with 15 targets

Among the 29 predicted targets, 9 were corroborated by CMM-related databases, including ETCM [15], HERB [41], and HIT [12] (Fig. 5A, B). Additionally, 3 targets were supported by published literature. For the predicted targets that were not annotated in these databases, molecular docking was performed for 17 targets. 15 targets showed stable binding interactions with baicalein, while docking failed for the remaining two (Fig. 5C). These results further confirmed the high confidence and predictive reliability of SYSTCM-TNet. The detailed validation results and GO/KEGG enrichment are provided in Fig S1. In summary, both database and literature evidence validated the reliability of SYSTCM-TNet, while molecular docking further confirmed its potential in discovering novel targets.

### Performance and algorithm combination analysis of the efficacies and properties model

We collected 616 CMMs from the *Chinese Pharmacopoeia*. After data standardization, 1561 CMM–efficacy associations and 2910 CMM–property associations were obtained. In addition, 616 CMMs and 40 pharmacological effects defined by our previous work [24] were used to perform 39,160 combinatorial queries in CNKI, yielding 2,555,485 literature records. Following binarization, 4,060 CMM–pharmacological effect associations were derived (Fig S2). A CMM-wise normalization analysis using the Q60 cutoff generated 4,229 positive associations and showed high consistency with the original matrix, with a pharmacological-effect-level Pearson correlation of 0.981 and a cell-wise agreement of 89.3%. (Supplementary Fig S3). In total, 33 binary classification datasets were constructed to cover efficacies, the five flavors, and meridians, together with one ternary classification dataset representing the cold–hot–neutral properties. For the quality-control compounds listed in the *Chinese Pharmacopoeia*, 207,283 literature records were retrieved, leading to the identification of 363 unique compounds associated with 1758 pharmacological effect relationships.

**Fig. 6:**
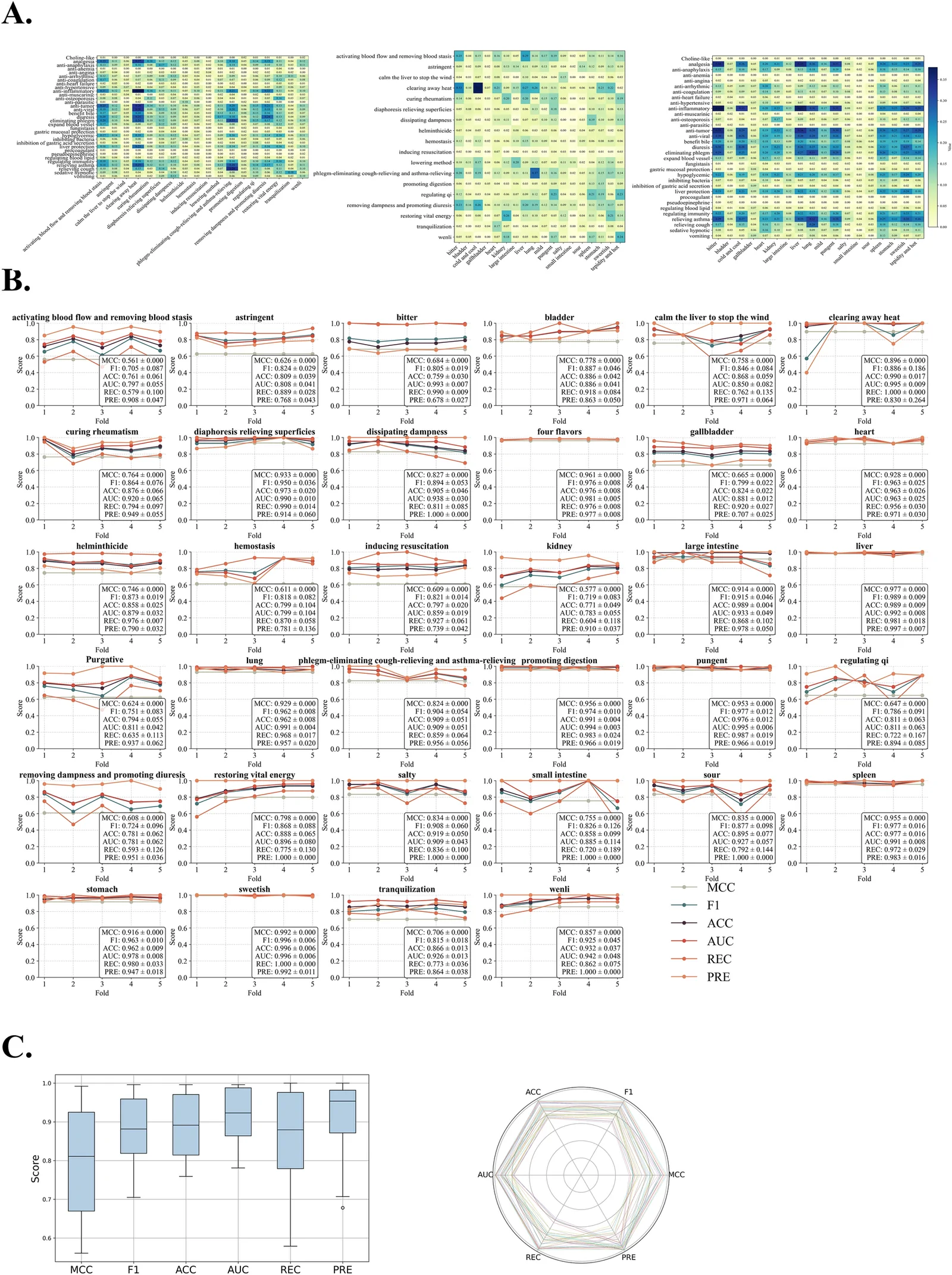
Jaccard analysis of dataset and model performance of the efficacy and property prediction models. **A** Jaccard correlation of pharmacological effects, efficacies, and CMM properties. **B** Model performance (MCC, F1, ACC, AUC, PRE, REC) was evaluated using 5-CV. **C** Performance visualization using boxplots and radar charts

Jaccard analysis was employed to quantify the correlations among pharmacological effects, properties, and efficacies using the CMMs matrix. The results revealed relatively strong associations (Fig. 6A), including anti-inflammatory effects with the efficacy of *clearing heat* (0.32 vs. an average of 0.05 for pharmacological effects–efficacies), anti-tumor effects with the *bitter* property (0.35 vs. an average of 0.07 for pharmacological effects–properties), and the *cold* property with *clearing heat* (0.51 vs. an average of 0.08 for efficacies–properties). The current study highlights that the bitter receptor induces apoptosis in multiple cancers [42–44], which is consistent with the Jaccard analysis in our study. Notably, the Jaccard between the efficacy of *wenli* and the *cold* property was 0, further supporting that efficacies and properties are mutually constrained and exhibit internal logical consistency within CMM theory.

Within the Optuna-based AutoML framework, 168 algorithmic combinations integrating feature selection, resampling, and classification methods were evaluated to build predictive models of properties and efficacies. Five-fold cross-validation demonstrated robust and consistent classification performance (Fig. 6B). Across the 34 classification tasks, the mean MCC (Fig. 6C) was 0.794, with the *sweetish* model achieving the highest score (0.992) and the model for *activating blood flow and removing blood stasis* yielding the lowest (0.705). The mean AUC reached 0.799, again with *sweetish* attaining the highest value (0.996), while the model for *removing dampness and promoting diuresis* exhibited the lowest (0.781). To ensure that models trained on the CMM dataset could accurately generalize to compound-level predictions, we imposed a constraint minimizing the absolute difference L between the positive-label distributions in CMMs and those in the predicted compounds. The resulting L averaged 0.072 (range: 0.002–0.277).

**Fig. 7:**
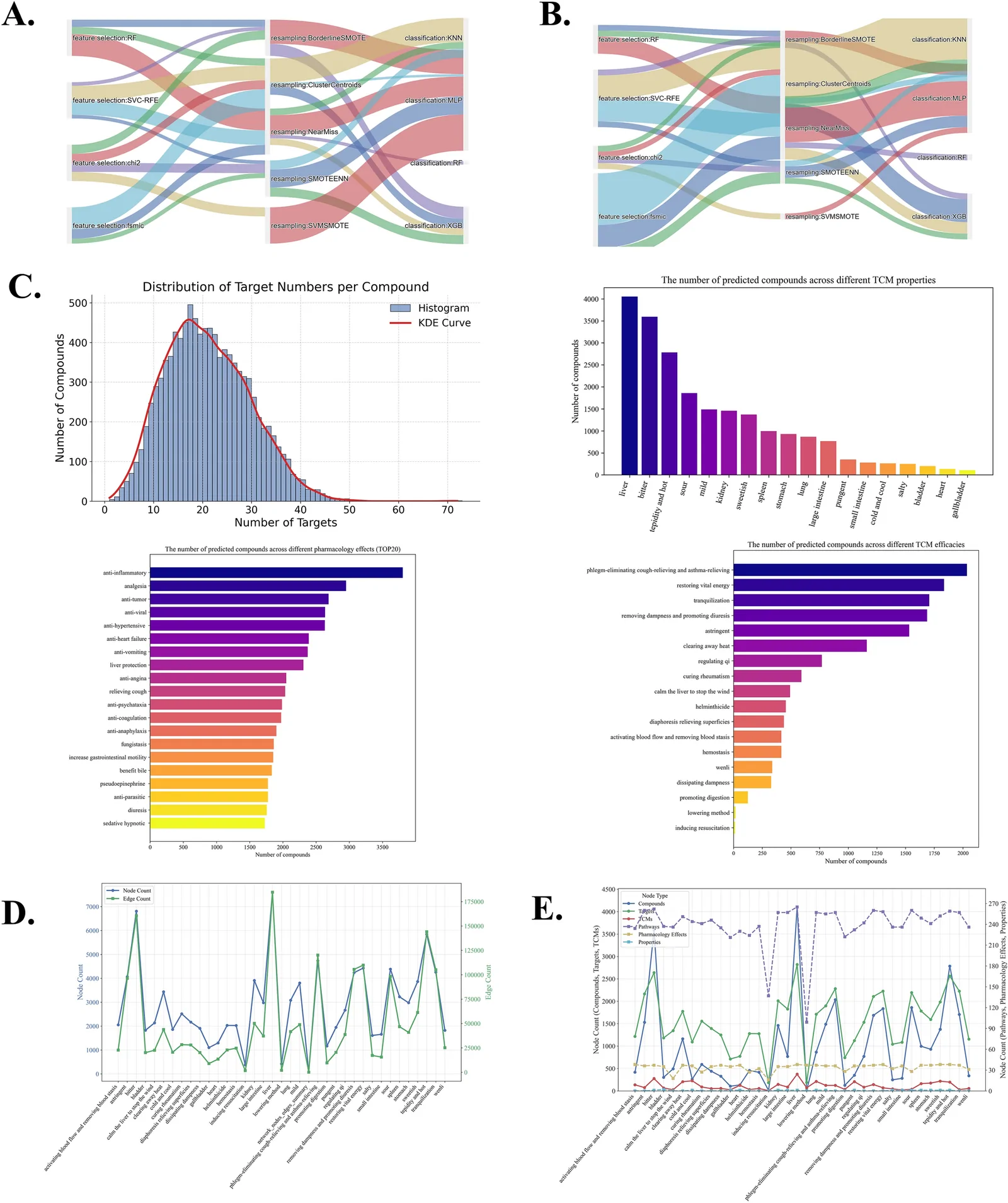
Evaluation of algorithm combinations, large-scale prediction results, and biological network summaries for CMM efficacies and properties. **A** Confidence analysis of different algorithm combinations. **B** Support analysis of different algorithm combinations. **C** Large-scale prediction of associations among compounds and targets, pharmacological effects, CMM efficacies, and CMM properties. **D** Total numbers of nodes and edges in 36 networks incorporating CMM efficacies and properties. **E** Numbers of nodes per type for each of the 36 networks incorporating CMM efficacies and properties

The frequency of the optimal algorithmic combinations is summarized in Fig S5. An association-rule analysis was then conducted to characterize typical patterns linking feature selection methods, resampling strategies and classifiers (Fig. 7A and B). Among feature selection–resampling pairs, the FSMIC–ClusterCentroids rule exhibited the strongest association, indicating that approximately 26% of all evaluated pipelines adopted this combination and that ClusterCentroids was chosen in 60% of models using FSMIC. For resampling–classifier pairs, ClusterCentroids–KNN was the most prominent rule, suggesting that nearly one third of all pipelines relied on the ClusterCentroids–KNN combination and that KNN was selected in two thirds of the models built with ClusterCentroids.

### FuTCM-PDD dataset and construction of biological networks based on property–efficacy associations

The FuTCM-PDD dataset comprised 7141 CMM herbs, 30,731 compounds, 4321 targets, 40 pharmacological effects, 18 properties, and 18 efficacies (Fig. 7C). Applicability-domain mapping further showed that most compounds analyzed in this study fell within SYSTCM-TNet domain, supporting the use of SYSTCM-TNet for subsequent target-prioritization analysis, as detailed in the Supplementary Information section “Most Chinese Materia Medica Compounds Fall within the DrugBank-Defined Chemical Applicability Domain.” Target prediction yielded 629,910 compound–target associations, with most compounds predicted to interact with 10–30 targets. Across 21,751 compound–property associations, *“liver”* and *“bitter”* were linked to the largest number of compounds. Among 65,151 compound–pharmacological effect associations, *“anti-inflammatory”*, *“analgesia”* and *“anti-tumor”* emerged as the dominant categories. In addition, 78,478 herb–compound associations were constructed. For CMM efficacies, 14,355 compound–efficacy associations were identified, with “*phlegm-eliminating cough-relieving and asthma-relieving*”, “*restoring vital energy*” and “*tranquilization*” most frequently represented.

To ensure accessibility, all data were organized in a public Git repository (https://github.com/SYSTCM/FuTCM-PDD) as six entity tables and five relationship tables. The dataset is fully open-access, with one-click retrieval functions to facilitate downstream applications such as network pharmacology analysis and knowledge graph construction.

The biological relevance of the property–efficacy associations was further evaluated by network randomization. By systematically integrating CMMs, compounds, targets, pathways, and pharmacological effects, we constructed 36 property- and efficacy-specific networks comprising a total of 106,240 nodes and 1,856,440 edges (Fig. 7D). Among these, the *liver* network contained the largest number of nodes and edges (7603 and 184,622, respectively), consistent with liver being the most frequently annotated CMM property category (Fig. 7E). In contrast, the *inducing resuscitation* network was the smallest (377 nodes and 1,649 edges), and this efficacy likewise involved the fewest CMMs (Fig S2). These concordant patterns between network topology and CMM category frequencies provide preliminary support for the biological plausibility of our network construction.

### Biological significance of property–efficacy networks by randomization

**Fig. 8:**
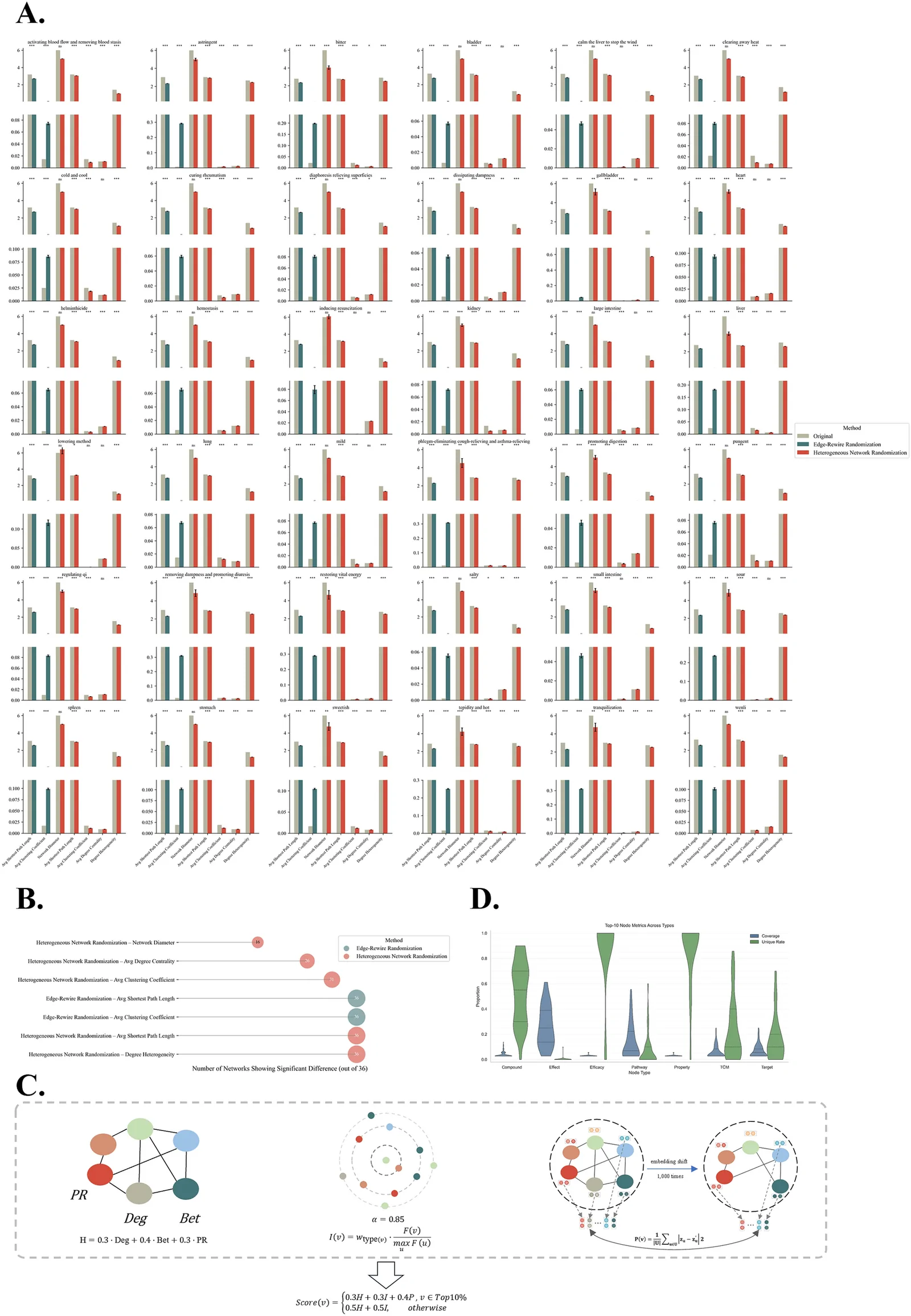
Topological significance of the 36 property–efficacy networks. **A** Each network was compared with two null models—degree-preserving edge-rewire and type-preserving heterogeneous randomization **B** Number of subnetworks (out of 36) showing significant (*P* < 0.05) deviation for each metric under the two randomization schemes. **C** Identification of key CMMs, compounds, and targets linked to properties and efficacies. **D** Coverage and uniqueness of the top 10 nodes across different categories

Considering different network ensembles requiring distinct combinations of topological metrics to adequately capture structural variation [45], we adopted two randomization strategies with tailored metric sets. For the edge-rewired ensemble, which preserves the node-degree sequence while randomizing the edges, we evaluated the average shortest path length and the clustering coefficient as well-established measures of global connectivity and local clustering. In contrast, for the heterogeneous ensemble, which perturbs both node-type composition and connectivity patterns, we additionally assessed network diameter, average degree centrality and degree heterogeneity to capture broader structural changes, including hub-related effects and degree diversity (Fig. 8A). For the edge-rewired ensemble all 36 property/efficacy-specific networks exhibited significant differences (Fig. 8B). For the heterogeneous ensemble, the average clustering coefficient, average degree centrality, and network diameter were significant in 31, 26, and 16 networks, respectively. These results collectively demonstrated that the property/efficacy-specific networks are not random structures but instead capture biologically meaningful structures. The consistent significance of shortest-path length across all networks highlights the presence of non-uniform connectivity patterns and efficient information flow, which are key features of functional biological networks [46]. Moreover, clustering coefficient, degree and network diameter revealed substantial variation among efficacy/property networks in their modular structure, dependence on hub nodes, and global compactness [47].

To quantitatively characterize each node within property/efficacy networks, we assigned scores by integrating static centrality, dynamic information flow, and perturbation sensitivity (Fig. 8C). The *clearing away heat* network was selected as a case study for further validation. The top-10 compounds and targets were subsequently validated through comprehensive literature mining. As direct experimental verification of CMM efficacies remains challenging, we performed a prior Jaccard analysis across a wide range of CMMs, which revealed strong associations between the efficacy of “*clearing away heat*” and pharmacological activities such as anti-inflammatory, anti-viral, anti-tumor, and analgesic effects. Literature retrieval in PubMed using combinations of “compounds/targets” and the corresponding pharmacological effects showed that the 8 targets of 10 and 9 compounds of 10 exist the literature evidence with one or more pharmacology effects (Table 3). This demonstrated that the network could capture meaningful biological and pharmacological associations, thereby linking traditional efficacy with modern pharmacological activities.

**Table 3:** Validation of the “*clearing away heat*” network through Top-10 compounds and targets

No	Targets			Compounds		
	Name	Effect	Validation	Name	Effect	Validation
1	MAPK12	Anti-inflammator; Anti-tumor	16947383^*^ 30971822^*^	Oleic acid	Anti-inflammatory; Anti tumor	33614661^*^; 39421735^*^
2	MAPK13	Anti-inflammatory; Anti-tumor	11068410^*^	Harmine	Anti-inflammatory; Anti tumor	40156005^*^; 39954215^*^
3	NDUFA4	Anti-inflammatory; Anti-tumor	38917002^*^40592341^*^	Sulforaphane	Anti-inflammatory	38007430^*^
4	MAP2K2	Anti-inflammatory; Anti-tumor	9116354^*^; 23629727^*^	Picric acid	–	–
5	UQCRC1	–	–	Maleic anhydride	Anti-virus; Anti-inflammatory; Anti-tumor	25690799^*^; 18522430^*^
6	PRKCB	Anti-inflammatory; Anti-viral	4693901^*^; 4138286^*^	AT38698	Analgesia	JP-7095868-B2 (Patent)
7	CHRNB2	Analgesia	20371741^*^	Paeonol	Anti-inflammatory; Anti-tumor	39117680^*^; 32626954^*^
8	GRIN3B	Analgesia	6482077^*^	Tetrandrine	Anti-tumor; Anti-virus;	39059614^*^; 37923171^*^
9	GLRA2	Analgesia	11105467^*^	Pyridoxal	anti-virus; Anti-tumor	6198801^*^; 35405490^*^
10	KCNC3	–	–	Ginsenoside rg1	Anti-tumor; Anti-inflammatory	24761846^*^; 31709615^*^

^*^represents the PMID in PubMed

To evaluate the reliability of the identified nodes in the biological networks, we calculated the coverage and uniqueness rates for each network. Coverage reflects the proportion of node types appearing across multiple networks, whereas uniqueness represents the proportion of node types that occur exclusively in a single network. The top 10 nodes within each category exhibited distinct patterns in terms of coverage and uniqueness rate (Fig. 8D). Since the 36 networks were constructed around specific properties or efficacies, these nodes inherently serve as network “labels” and thus exhibit strong network-specificity, resulting in high uniqueness. In contrast, compounds, CMMs, and targets are often shared across multiple networks, thereby maintaining a balance between coverage and uniqueness. Within each category, the networks contain both specific nodes and important nodes shared with other networks. Pathways and pharmacological effects, representing higher-level biological functions, are more likely to be shared across multiple networks. Consequently, they exhibit higher coverage, but lower uniqueness compared with other categories.

### Experimental validation of FuTCM-PDD predictions: phenotypic-efficacy–driven discovery of a novel lipid-lowering candidate

Hyperlipidemia is a major contributing factor to the development of multiple diseases, including diabetes and other metabolic disorders [48, 49]. To investigate whether integrating phenotypic efficacy information can guide the discovery and mechanistic interpretation of anti-hyperlipidemic CMMs, we first applied Jaccard analysis and found that the efficacy term *“dissipating dampness”* was most strongly associated with *“regulating blood lipids”* (Jaccard index = 0.073, approximately seven-fold higher than the average value). Based on this finding, we used the efficacy term as a phenotypic handle to mine the network: we collected all compounds predicted to possess the efficacy of *“dissipating dampness”* and mapped them to their corresponding CMMs. Among these, NRR contained the largest number of such compounds. NRR is a classical CMM traditionally used for the treatment of knee bone pain, such as rheumatoid arthritis. To the best of our knowledge, no previous study has reported its role in regulating blood lipids.

**Fig. 9:**
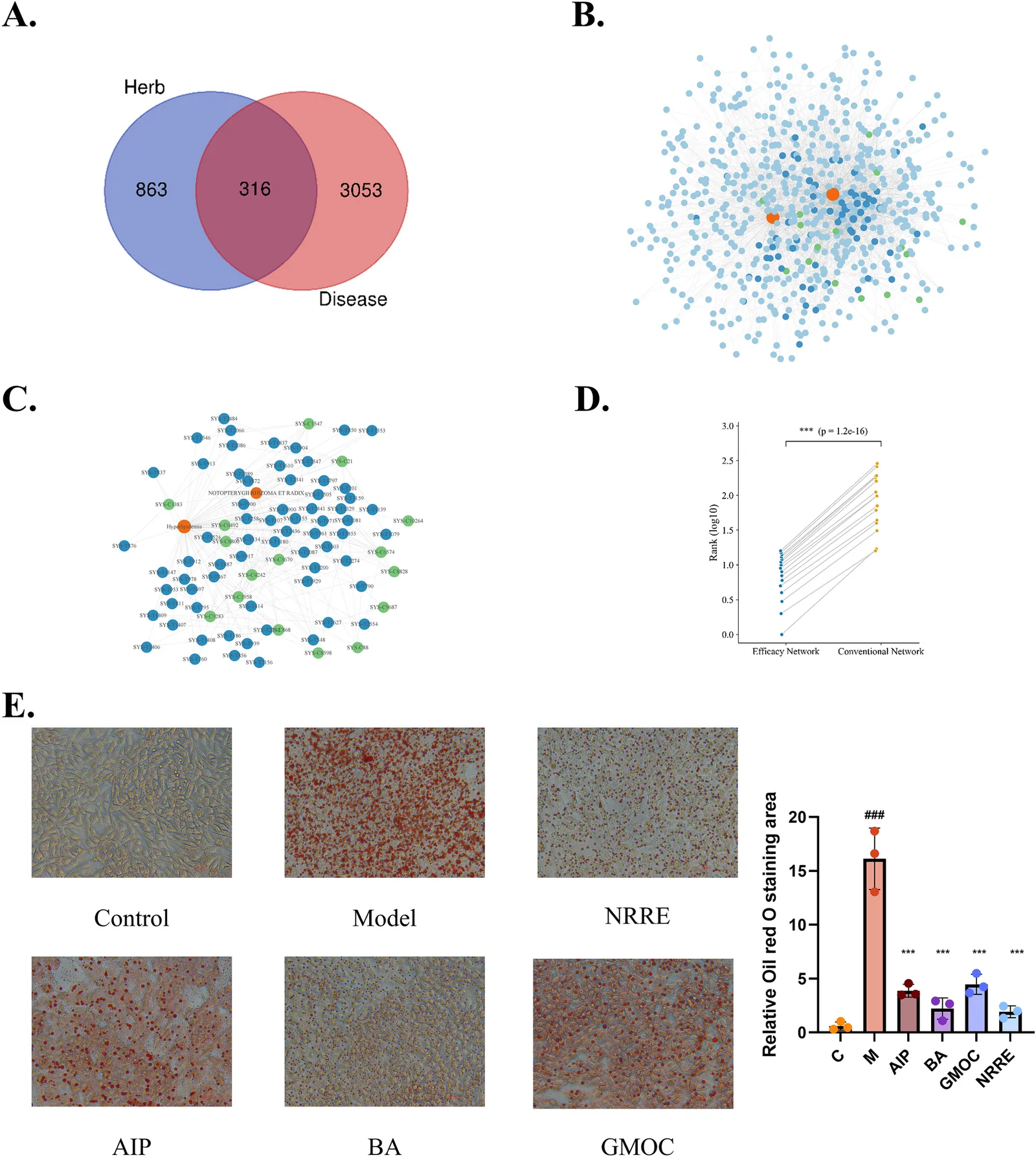
FuTCM-PDD identifies NRRE and its compounds as candidate phenotypic therapies for hyperlipidemia. **A** The overlap of targets between NRR and hyperlipidemia. **B** Conventional network analysis of NRR for hyperlipidemia. **C** Phenotypic efficacy–driven network analysis of NRR for hyperlipidemia. **D** Comparative ranking of active constituents by degree centrality in the conventional versus phenotypic efficacy-driven network. **E** Oil Red O staining (representative, 40X magnification) of oleic/palmitic acid-induced HepG2 cells treated with NRRE, AIP, BA, and GMOC

In parallel, we compiled target sets for NRR and hyperlipidemia. The intersection of these targets (Fig. 9A), together with their associated compounds, was used to construct the conventional network. We then overlaid phenotypic efficacy information and constructed an efficacy-driven network by further filtering constituents annotated with the efficacy of *dissipating dampness*. This design allowed a direct comparison between standard network pharmacology and our phenotypic efficacy–integrated framework in prioritizing candidate compounds. Degree analysis in the conventional network pharmacology approach indicated that oleic acid, osthole, retinol, D-limonene, and linoleic acid were the top five candidates (Fig. 9B). These compounds are widely present in various CMM herbs and are used to treat multiple diseases, illustrating the well-recognized tendency of conventional network pharmacology to prioritize ubiquitous, non-specific metabolites. In contrast, the efficacy-driven network pharmacology approach identified more specific active components, including 5-geranoxy-7-methoxycoumarin, alloisoimperatorin, bornyl acetate, xanthotoxol, and osthenol among the top five candidates (Fig. 9C), and these compounds exhibited marked upward shifts in degree ranking relative to the conventional network (Fig. 9D), indicating that incorporation of phenotypic efficacy information can re-rank and highlight more specific constituents. Additional KEGG comparison showed that lipid-related pathways were preferentially ranked in the efficacy-driven network compared with random same-size compound networks (Supplementary Fig. S6). Functionally, we established a lipid-overload model in HepG2 cells and treated cells with NRR, AIP, BA, and GMOC at their maximum non-cytotoxic concentrations (Supplementary Fig S7). Oil Red O staining confirmed that the constituents prioritized by the efficacy-driven network reduced intracellular lipid accumulation (Fig. 9E). We further performed RT-qPCR validation of two genes: FABP1, a key target within the efficacy-driven network, and CYP51A1, a lipid-metabolism-related downstream gene not included in the network. The expression levels of both genes were reduced in the oleic acid/palmitic acid-induced HepG2 model, whereas BA treatment significantly reversed these reductions (Supplementary Fig. S4). Collectively, these results demonstrate that integrating phenotypic efficacy information into the network framework mitigates the long-recognized homogeneity of conventional network pharmacology, and cell-based assays further corroborate the pharmacological activity of the specific-compounds.

## Discussion

Phenotype-based screening has recently gained considerable attention owing to its relatively high clinical success rate in drug discovery [50]. However, systematic strategies for phenotype-based drug discovery remain insufficiently developed. In CMM, extensive phenotypic data have been historically codified in the form of classical efficacy theories. Fusing these classical insights with modern phenotype-based approaches not only deepens the understanding of CMM theory but also harnesses traditional wisdom to guide and enrich contemporary drug discovery. Here we present FuTCM-PDD—the first comprehensive framework for decoding the properties and efficacies of CMMs to enable phenotype-based drug discovery. The DTI module, built upon KAN, together with the efficacy–property association module based on AutoML, provides a solid data foundation for the full-chain integration of CMM knowledge encompassing CMM herbs, compounds, targets, pharmacological effects, efficacies, and properties. Leveraging large-scale multilayer data, we constructed a property–efficacy network whose biological relevance was validated through heterogeneous network randomization and edge-rewiring analyses, thereby enabling the quantitative representation of CMMs, compounds, targets, pathways, pharmacology effects, CMM properties, and efficacies.

Network pharmacology has been widely applied to elucidate mechanisms of action and to discover novel active ingredients in CMM, owing to its compatibility with the multi-compound, multi-target characteristics. However, two major limitations remain: first, conventional network pharmacology requires extensive data collection, which is labor-intensive and highly repetitive [51, 52]. In FuTCM-PDD, we address this limitation by releasing complete, standardized raw datasets covering all CMM herbs, compounds, targets, pharmacological effects, efficacies, and properties, thereby enabling researchers to directly process, extract, and extend these resources without repeating basic data-mining steps. Second, the issue of homogeneity in network pharmacology is increasingly recognized [53]. Rather than solely relying on topological features in compound–target networks, our strategy improves node selection by integrating CMM-theoretical consistency at the phenotype level and excluding efficacies that are inconsistent with CMM theory for the disease. Coupled with more accurate predictions from the KAN-based DTI module and the AutoML-derived efficacy–property models, this refinement substantially alleviates the tendency of networks to be dominated by ubiquitous compounds and broadly acting targets. The NRR–hyperlipidemia case study exemplifies these methodological advantages. By exploiting the strong association between the efficacy term *dissipating dampness* and the phenotype of *regulating blood lipids*, FuTCM-PDD prioritized more specific candidate constituents of NRR, in contrast to conventional network pharmacology, which tended to highlight widely distributed metabolites. Importantly, the screening results for NRR and its candidate compounds were preliminary validated in oleic/palmitic acid-induced HepG2 cells. FuTCM-PDD provides a novel computational perspective for prioritizing potential active compounds from CMM, while also serving as a valuable complement to conventional network pharmacology.

There are several avenues for further investigation. For the compound–target interaction module, the current SYSTCM-TNet is more suitable for target prioritization of compounds located within the DrugBank-defined chemical applicability domain and within a known candidate target space. Its generalization ability for out-of-domain compounds and completely unseen targets still requires further improvement. In future work, larger cross-database DTI resources, more comprehensive target functional annotations, and graph-based contrastive learning will be incorporated to improve model robustness and external generalizability [54]. For the efficacy–property prediction model, the limited number of samples and the high complexity of sample features continue to hinder the mechanistic interpretation of CMM theory. In addition, defining pharmacological effect associations using the criterion of publication count > 10 may introduce potential label bias, and future studies may adopt more comprehensive evidence-weighting strategies, such as hybrid criteria combining minimum publication counts, herb-wise normalized proportions, and evidence-quality weights. Integrating pharmacological features derived from high-throughput transcriptomic data could provide a more accurate characterization of the biological functions of CMMs [55]. The relationships between individual compounds and herb-level efficacies and properties remain complex and require further experimental and mechanistic evidence for more reliable modeling. Although NRRE and the candidate compounds reduced lipid accumulation in HepG2 cells under non-cytotoxic conditions, these results do not establish clinically achievable exposure, in vivo efficacy, or detailed lipid-regulatory mechanisms. Further studies are needed to quantify these compounds in raw NRR and NRR extract, evaluate pharmacokinetic exposure, and validate their lipid-lowering effects in vivo. At the same time, such integration would offer more interpretable medical features that align traditional CMM theories with modern biomedical understanding.

## Conclusion

We developed the first comprehensive phenotype-driven framework, FuTCM-PDD, for decoding CMM properties and efficacies to enable phenotype-based drug discovery. Built on a KAN-based DTI prediction model (SYSTCM-TNet) and an AutoML framework, FuTCM-PDD integrates multi-level information that CMMs, compounds, targets, pathways, pharmacological effects, efficacies, and properties, thereby providing a phenotype-oriented strategy to help reduce the recurrent prioritization of commonly reported associations in conventional network pharmacology. Using FuTCM-PDD, we preliminarily identified NRR as a candidate herb with a lipid-lowering phenotype, together with its potential active compounds. Overall, FuTCM-PDD provides an AI-assisted strategy for phenotype-oriented CMM research and offers a scalable computational perspective for transforming traditional medical knowledge into mechanism-informed candidate discovery.

## Supplementary Information

Supplementary Material 1.

Supplementary Material 2.

## Data Availability

All data supporting the findings of this study are available within the github (https://github.com/SYSTCM/FuTCM-PDD) and its Supplementary Information.

## Abbreviations

5-CV: Five-fold cross-validation
AIP: Alloisoimperatorin
AUC: Area under the receiver operating characteristic curve
AutoML: Automated machine learning
BA: Bornyl acetate
CMM / CMMs: Chinese Materia Medica
DCG: Discounted cumulative gain
DS: Discovery studio
DTI: Drug–target interaction
ECFP/ECFPs: Extended-connectivity fingerprint(s)
FM: Factorization machine
GCN: Graph convolutional network
GMOC: 5-Geranoxy-7-methoxycoumarin
KAN/KANs: Kolmogorov–Arnold network(s)
KNN: K-nearest neighbors
LR: Logistic regression
LSTM: Long short-term memory
MAP: Mean average precision
MCC: Matthews correlation coefficient
FSMIC: Mutual information coefficient
MLP: Multilayer perceptron
MRR: Mean reciprocal rank
NDCG: Normalized discounted cumulative gain
NRR: Notopterygii Rhizoma et Radix
NRRE: Notopterygii Rhizoma et Radix extract
PDP: Partial dependence plot(s)
RF: Random forest
ROC: Receiver operating characteristic
SVC-RFE: Support vector classifier–recursive feature elimination
SVM-RFE: Support vector machine–recursive feature elimination
SVM-SMOTE: Support vector machine–Synthetic Minority Over-sampling Technique
TCM: Traditional Chinese Medicine
