# Supplementary material for: FuTCM-PDD: a multi-level information fusion framework integrating KAN modeling and AutoML for phenotype-based drug discovery from Traditional Chinese Medicine
Source: Chin Med. 2026 Sep 21;21:220. doi: 10.1186/s13020-026-01490-1 (PMC13591747; doi:10.1186/s13020-026-01490-1)
Supplement: Supplementary file 1 [file 13020_2026_1490_MOESM1_ESM.docx]

# FuTCM-PDD: A Multi-Level Information Fusion Framework Integrating KAN Modeling and AutoML for Phenotype-Based Drug Discovery from Traditional Chinese Medicine

Zewen Wang ^a^, Yanxia Liu ^a^, Yue Ren ^a^, Qun Li ^a^, Jiaye Tian ^a^, Bin Yu ^a^,

Yuezhong Zhu ^a^, Jiaqi Wang ^a^, Miao Li ^a^, Liansheng Qiao ^a,*^, Yanling Zhang ^a,*^

^1^ Key Laboratory of TCM-information Engineer of State Administration of TCM, School of Chinese Materia Medica, Beijing University of Chinese Medicine, Beijing 102488, China

**Supplementary Methods:**

## Compound cold-start validation

To further evaluate the generalization ability of SYSTCM-TNet for unseen compounds, we performed an additional compound cold-start validation based on the DrugBank DTI dataset. In this setting, the compounds in the test set were completely absent from the training set, while all other procedures were kept consistent with those used in the random-split experiment. This setting better reflects the downstream application scenario of predicting potential targets for Chinese Materia Medica compounds.

Model performance was evaluated using Recall@10, MAP@10, MRR@10, and NDCG@10, and was compared with that obtained under the random drug–target interaction-pair split to assess the potential overestimation caused by random splitting.

## Performance of the ChEMBL external validation set

To evaluate the generalization ability of SYSTCM-TNet in an independent database, we constructed an external validation set based on ChEMBL. Human single-protein drug–target interaction records were retained, with compounds required to have standard SMILES information and targets required to have available protein sequence information. Drug–target pairs with pChEMBL ≥ 6 were defined as positive interactions[1]. To avoid information overlap between the DrugBank training data and the ChEMBL external validation set, drug–target interaction pairs already present in DrugBank were further removed.

Except for the source of positive samples, all remaining processing procedures for the ChEMBL external validation set were consistent with those used for the DrugBank dataset, including drug-side feature construction, protein-side feature construction, negative sampling, and ranking-based evaluation metrics. The trained SYSTCM-TNet model was directly applied to the ChEMBL external validation set without additional parameter updating.

## DrugBank input-space-based chemical applicability-domain analysis

Considering that external validation performance may be influenced by chemical-space differences between DrugBank and ChEMBL, we further performed a chemical applicability-domain analysis based on the DrugBank training drug input space.

First, the 2816-dimensional input vectors of DrugBank training drugs were used to fit the StandardScaler and PCA models, and DrugBank drugs, ChEMBL molecules, and Chinese Materia Medica compounds were projected into the same PCA50 feature space. Next, for each DrugBank training drug, the Euclidean distance to its nearest DrugBank training neighbor was calculated, generating the internal nearest-neighbor distance distribution of DrugBank. The 99th percentile of this distribution was used as the threshold defining the DrugBank 99% chemical applicability domain[2]. For each molecule in the external validation set or Chinese Materia Medica dataset, its Euclidean distance to the nearest DrugBank training drug was calculated in the same PCA50 space. Molecules with distances not exceeding this threshold were considered to fall within the DrugBank 99% chemical applicability domain, whereas those exceeding the threshold were considered outside the applicability domain. UMAP was used only to visualize the overall distribution of DrugBank, ChEMBL, and Chinese Materia Medica molecules in the DrugBank-centered input space, and was not used for applicability-domain assignment.

## Normalization analysis of Chinese Materia Medica–pharmacological effect associations

To assess the potential influence of research-volume bias on the construction of the Chinese Materia Medica–pharmacological effect association matrix, we performed an additional normalization analysis. For each Chinese Materia Medica *i* and pharmacological effect *j*, a proportional score was calculated by dividing the publication count of the corresponding Chinese Materia Medica–pharmacological effect pair by the total publication count of Chinese Materia Medica *i* across all pharmacological effects.

To binarize the normalized scores, the distribution of all non-zero herb-wise proportional scores was examined, and the 60th percentile was selected as the cutoff. Chinese Materia Medica–pharmacological effect pairs with proportional scores greater than or equal to this cutoff were encoded as positive associations.

To evaluate the consistency between the original publication count–based matrix and the normalized matrix, comparisons were performed at two levels. At the pharmacological-effect level, the number of associated Chinese Materia Medica was counted for each of the 40 pharmacological effects under both methods, and Pearson correlation analysis was used to evaluate consistency in pharmacological-effect coverage. At the cell level, the proportion of unchanged binary labels across all Chinese Materia Medica–pharmacological effect pairs was calculated as the cell-wise agreement.

## Same-size randomization analysis to control the funnel effect

To evaluate whether the efficacy network showed preferential enrichment of lipid-related mechanisms, we performed KEGG pathway enrichment analysis using the target genes of the dissipating-dampness-associated compounds in NRR. The targets associated with the treatment of hyperlipidemia by the *dissipating-dampness*-related compounds were then subjected to KEGG pathway enrichment analysis using Enrichr through gseapy.

Four lipid-related pathways were selected for focused comparison: lipid and atherosclerosis, arachidonic acid metabolism, linoleic acid metabolism, and fluid shear stress and atherosclerosis. To address whether the observed pathway prioritization was merely a funnel effect caused by reducing the network, we performed a same-size randomization control. Specifically, we randomly sampled 1,000 compound sets from the NRR compound pool, with each random set containing the same number of compounds as the *dissipating-dampness* efficacy network. The same KEGG enrichment workflow was applied to each random set using its corresponding compound targets.

Pathways were ranked according to their enrichment *P* values. For the four lipid-related pathways, we compared their rank percentiles in the dissipating-dampness efficacy network with the average rank percentiles obtained from the random compound networks. Lower percentile values indicate higher pathway prioritization. This analysis was used to assess whether lipid-related pathways were preferentially ranked in the efficacy-guided network compared with random same-size compound networks.

## Cell culture and viability assay

HepG2 cells were maintained in DMEM supplemented with 10% FBS and 1% penicillin–streptomycin at 37 °C in 5% CO₂. CCK8 was used to measure the absorbance at 450 nm wavelength. The cell survival rates are calculated as [(ODdrug-ODblank)/ (ODcontrol-ODblank)] × 100 %.

## RT-qPCR

Total RNA was extracted from cultured cells using an RNA extraction kit containing an RNase-free spin column CR3 (Tiangen, Beijing, China). The extracted RNA was reverse-transcribed into cDNA using the FastKing RT Kit with gDNase (Tiangen, Beijing, China). RT-qPCR was performed using TB Green® Premix Ex Taq™ (TaKaRa, Japan). Relative mRNA expression levels were calculated using the 2^−ΔΔCt method, with GAPDH serving as the internal reference gene. The primers were synthesized by Sangon Biotech (Shanghai, China), and their sequences are listed in Table S1.

Table S1 Primer sequences used for RT-qPCR

| Gene Name | Forward Primer | Reverse Primer |
| --- | --- | --- |
| GAPDH | GGAGCGAGATCCCTCCAAAAT | GGCTGTTGTCATACTTCTCATGG |
| FABP1 | GTGTCGGAAATCGTGCAGAAT | GACTTTCTCCCCTGTCATTGTC |
| CYP51A1 | AGGCGATGGAGAAGGTGAC | GTATGGAGGACTTTTCACCC |

**Supplementary Results**

## Compound cold-start validation indicates that the model retains target-ranking ability for unseen compounds

Under the original random drug–target interaction-pair split, SYSTCM-TNet achieved Recall@10, MAP@10, MRR@10, and NDCG@10 values of 0.4631, 0.2394, 0.2418, and 0.2880, respectively. Under the stricter compound cold-start setting, model performance decreased, with Recall@10, MAP@10, MRR@10, and NDCG@10 values of 0.2252, 0.0952, 0.1281, and 0.1358, respectively (Table S2).

These results indicate that random interaction-pair splitting may indeed overestimate model performance; nevertheless, SYSTCM-TNet still retained a certain degree of target-ranking ability when the test compounds were absent from the training set.

Table S2 Internal validation under random and compound cold-start data splits

| Model | Data setting | RECALL@10 | MAP@10 | MRR@10 | NDCG@10 |
| --- | --- | --- | --- | --- | --- |
| SYSTCM-TNet | Random split | 0.4631 | 0.2394 | 0.2418 | 0.2880 |
| SYSTCM-TNet | compound cold-start | 0.2252 | 0.0952 | 0.1281 | 0.1358 |

## ChEMBL external validation reveals that model generalization is affected by the chemical applicability domain

On the full ChEMBL external validation set, SYSTCM-TNet achieved Recall@10, NDCG@10, MAP@10, and MRR@10 values of 0.1675, 0.1079, 0.0827, and 0.0995, respectively (Table S3). This performance was lower than that observed in the DrugBank internal random-split test, suggesting a clear distributional difference between the ChEMBL external validation set and the DrugBank training data.

To further interpret this observation, ChEMBL external molecules were stratified according to the DrugBank 99% chemical applicability domain. Among 474,393 ChEMBL external molecules, 12,598 molecules were located within the DrugBank 99% chemical applicability domain, accounting for 2.66% of the full ChEMBL external set. Molecules within this applicability domain showed markedly better ranking performance, with Recall@10, NDCG@10, MAP@10, and MRR@10 values of 0.2735, 0.1946, 0.1621, and 0.1828, respectively. In contrast, molecules outside the applicability domain achieved Recall@10, NDCG@10, MAP@10, and MRR@10 values of 0.1646, 0.1056, 0.0806, and 0.0973, respectively.

These results indicate that the generalization ability of SYSTCM-TNet in an external database is closely related to the coverage of the training chemical space. External molecules located within the DrugBank-centered input space showed better target-ranking performance, whereas molecules outside this chemical applicability domain showed reduced performance. Therefore, the lower overall performance on the full ChEMBL external set mainly reflects chemical-space differences between the external database and the DrugBank training set.

Table S3 External validation performance across the DrugBank-defined applicability domain

| ChEMBL subset | No. of molecules | RECALL@10 | NDCG@10 | MAP@10 | MRR@10 |
| --- | --- | --- | --- | --- | --- |
| Full ChEMBL set | 474,393 | 0.1675 | 0.1079 | 0.0827 | 0.0995 |
| Inside DrugBank 99% applicability domain | 12,598 | 0.2735 | 0.1946 | 0.1621 | 0.1828 |
| Outside DrugBank 99% applicability domain | 461,795 | 0.1646 | 0.1056 | 0.0806 | 0.0973 |

## GO and KEGG enrichment analysis of predicted baicalein targets

Baicalein has been reported to exert anti-cancer[3, 4], anti-inflammatory[5], and anti-Alzheimer’s disease activities[6]. We predicted candidate targets of baicalein and performed pathway enrichment analysis, which revealed pathways closely aligned with these pharmacological profiles (Fig. S1), including cancer-related pathways (Pathways in cancer, PD-1/PD-L1 checkpoint, viral carcinogenesis), inflammatory pathways (Th17 cell differentiation, inflammatory bowel disease, adipocytokine signaling), and mechanisms relevant to neurodegeneration and Alzheimer’s disease (AGE–RAGE signaling, insulin resistance, lipid and atherosclerosis, oxidative stress–related pathways). Previous studies have shown that blocking PD-1/PD-L1 interactions can enhance T-cell responses and mediate anti-tumor activity[7], that transdifferentiation of Th17 cells into regulatory cells promotes the resolution of inflammation[8], and that the AGE–RAGE axis acts as a driver of neurodegeneration, where inhibiting AGE–RAGE interactions can slow the progression of neurological disease[9, 10]. The concordance between our enrichment results and these established mechanisms supports the reliability of the target prediction for baicalein.

Fig. S1 GO and KEGG enrichment analysis of predicted baicalein targets. (A) Top 20 KEGG pathways. (B) Top 10 GO terms (with only 6 terms enriched in the cellular component category).

## Most Chinese Materia Medica compounds fall within the DrugBank-defined chemical applicability domain

After projecting Chinese Materia Medica compounds into the same DrugBank-centered PCA50 input space, 30,546 compounds with parsable SMILES and complete model input vectors were retained, whereas compounds whose SMILES could not be parsed by RDKit were excluded. Based on the DrugBank 99% nearest-neighbor distance threshold, 28,002 Chinese Materia Medica compounds were located within the DrugBank 99% chemical applicability domain, accounting for 91.67% of valid Chinese Materia Medica compounds. Only 2,544 compounds were located outside this applicability domain.

These results indicate that the downstream target prediction for Chinese Materia Medica compounds in this study was mainly performed within the chemical space covered by DrugBank training compounds. Therefore, the application of SYSTCM-TNet in this study should be interpreted as target prioritization for new Chinese Materia Medica compounds located within or near the DrugBank chemical applicability domain in a known candidate target space, rather than unrestricted prediction for arbitrary external molecules, completely novel targets, or samples outside the training distribution. Predictions for compounds located outside the applicability domain should be interpreted more cautiously and require further support from literature evidence, experimental validation, or additional models.

## Standardization and mapping of Chinese Materia Medica efficacy terms

Raw efficacy and property annotations were collected from the Chinese Pharmacopoeia, and external-use-only efficacies were excluded. The remaining efficacy descriptions were decomposed according to their verb–object semantic structure and manually checked to obtain original efficacy terms. These terms were then standardized with reference to *Efficacy of Chinese Materia Medica* and commonly accepted classification principles in Chinese materia medica and Chinese pharmacology. To reduce label sparsity and class imbalance, the original efficacy terms were further merged into 18 major efficacy categories. A conservative inclusion principle was applied during mapping: only clearly defined and widely accepted efficacy assignments were retained, whereas ambiguous, controversial, or highly overlapping terms were excluded from the corresponding modeling labels. The final mapping table was reviewed by CMM-domain experts and is provided in Supplementary Table S4.

Table S4 Mapping table for efficacy standardization

| Standardized efficacy category | Original efficacy term |
| --- | --- |
| tranquilization | 镇心安神，养心安神，宁心安神，安神益智，养心，宁心，养血安神，补肾安神 |
| restoring vital energy | 补血，养血，补血养血，养血补心，益肝,补阳，补火助阳，补肾阳，助阳，助阳化气，壮阳,补阴，养阴，益阴，滋阴,益精，补精，益精补髓，补益精血，益精填髓,滋养补虚，补气，补虚弱，补益心脾，补中 |
| dissipating dampness | 化湿，化浊，除湿，化湿浊，芳香化湿,健脾，健运脾胃，补脾，辟秽 |
| phlegm-eliminating cough-relieving and asthma-relieving | 利咽喉，利咽开音，解毒利咽,平喘，定喘，宣肺平喘，纳气平喘，降气平喘，化痰，化饮,祛痰，消痰，涤痰，开痰，坠痰，豁痰，祛痰，温化寒痰，清化热痰,泻肺，泻肺气，泻肺火,镇咳，宣肺止咳，止咳,利气，除痰,润肺、润燥、润肺燥、润滑燥,宣肺、宣肺气、宣开肺气、开宣肺气、清宣肺气、宣肺疗哑、宣肺利咽、宣肺发汗、宣散肺经风热 |
| calm the liver to stop the wind | 平肝潜阳，潜阳，平抑肝阳，平肝阳，镇肝，息风，息肝风，止痉，平肝息风，镇肝息风，祛风解痉，解痉 |
| diaphoresis relieving superficies | 发汗解表，发汗解肌，发表散风，解表散风，散寒祛风，发散风寒，发散风热，祛风散寒发表，解表，疏散风热，疏散，解肌退热，疏散退热，疏风，通窍，通鼻窍，宣通鼻窍，宣散风热 |
| inducing resuscitation | 开窍醒神，利心窍，利窍 |
| regulating qi | 理气，行气，破气，和中,降气、下气、降肺气、清降肺气、肃降肺气、降逆、降止逆之肺气,宽中、宽中除胀、畅中，除满，止呃 |

Table S4. (Continued) Mapping table for efficacy standardization

| Standardized efficacy category | Original efficacy term |
| --- | --- |
| clearing away heat | 清热泻火，清热，泻火，清泻胃火，清泻肺火，清肺泄热，清肺泻火，清心，清心火，清心降火，降火，清火，清心泻热，清肺，清肺热(火)，清肝火，清肝，清肝泻火，清泄肝火，清肝热，清泻肝热，清降肝火，泻肝胆火，清泻胃热，清胃火，清胃热，降热，退虚热，清虚热，除骨蒸，除疳热，除烦热，清热毒，清胃热，清湿热，清利湿热，除蒸，除热，清热结，退虚热，滋阴降火，清肺降火 |
| removing dampness and promoting diuresis | 利水，利尿，利小便，利水消肿，利尿消肿，行水消肿，利水渗湿，利水湿，利湿，渗湿，利湿热，清利湿热，利水通淋，利尿通淋，利湿通淋，通淋，通利小便，利水道，渗湿泄热，宣肺利尿，清利肝胆湿热,排石，化石，排出结石,退黄疸，利胆退黄，祛湿退黄 |
| helminthicide | 防腐，化腐，去腐,疗癣，截疟，灭虱,杀虫、安蛔，驱虫 |
| curing rheumatism | 强筋健骨，强腰膝，强骨，活络，强筋骨,祛风除湿，胜湿，祛风，祛外风，祛风通弊，除弊，散寒通弊，散寒祛风，祛风散寒，去湿除弊,温补肝肾，补益肝肾，益肝肾，补养肝肾，补肾养肝，益肾养肝，补肝肾阴，利关节，祛风，祛外风，祛风，祛风湿,续筋接骨、续伤、接骨、续折，续折伤 |
| astringent | 固摄，固脱，固肾，敛肺气，固崩,清热止痢，凉血止痢,收敛止带，燥湿止带,止汗，收敛止汗，敛汗固脱，固表止汗,止泻，收涩止泻，涩肠固脱，固精、涩精、固精止遗、收敛固摄，摄唾，摄血，止崩，止崩漏，止泻痢,缩尿、止遗、缩尿止遗、止遗溺、固摄、收敛固涩 |
| wenli | 回阳通脉，回阳救逆，散寒，温中，温心阳，温肺，温脾阳，暖脾，温脾，温胃，温肾，温肾暖肝，温肾散寒，温肾阳，暖肾，温经，祛寒，温中散寒，引火归元 |

Table S4. (Continued) Mapping table for efficacy standardization

| promoting digestion | 消积，化积，去积，消积滞，消积导滞，导滞，消食化滞,健胃,开胃，开胃进食，进食，消食，消食化积，消食和胃，消化油腻肉食积滞，促进淀粉性食物消化，制酸，收敛制酸 |
| --- | --- |
| purgative | 软坚，润下软坚，泻下攻积，泻下，泻下通便，泻下逐水，泻水逐饮，泻下导滞，逐水，逐水退肿，峻下冷积，润肠通便，滑肠通便，润肠，滑肠，润肠燥，通便，治便秘，温阳通便，缓泻，轻泻，通利二便 |
| hemostasis | 收敛止血，固经止血，化瘀止血，温经止血，散瘀止血，凉血止血 |
| activating blood flow and removing blood stasis | 活血，祛瘀，化瘀，散瘀，破瘀，破血，逐瘀，活血祛瘀，活血散瘀，破血逐瘀，通利血脉，行血脉，通血脉，利血脉，和血，活血调经，活血通经，调经止痛，养血调经，通经，通经止痛，化癥，活血化瘀 |

## Summary of CMM properties, pharmacological effects, and efficacies

The number of CMM herbs associated with analgesia (355), anti-inflammatory activity (474), relieving asthma (340), eliminating phlegm (306), anti-viral activity (300), and anti-tumor activity (401) is relatively large (Fig. S2). These effects are closely related to the treatment of common diseases: analgesia and anti-inflammatory effects are widely used across multiple systems[11, 12], whereas relieving asthma and eliminating phlegm are characteristic advantages of CMM in respiratory disorders[13]. Accordingly, these domains have accumulated richer literature and data. By contrast, pharmacological categories such as cholinomimetics (2), pseudoepinephrine (1), procoagulant (1), and anti-anemia (3) involve far fewer herbs, possibly reflecting lower clinical demand, methodological limitations, or insufficient experimental evidence.

Among CMM property categories, herbs for clearing away heat (249) are the most abundant, whereas those for inducing resuscitation (12) are the fewest. Anti-inflammatory activity corresponds to the largest herb set, consistent with our Jaccard analysis indicating a strong association between clearing away heat and anti-inflammatory effects. In terms of the four Qi, cold and cool herbs (266) slightly outnumber tepidity and hot herbs (215). For the five flavors, bitter herbs (315) predominate, while sour (42) and salty (54) herbs are relatively scarce. Regarding meridian tropism, herbs entering the liver meridian (308) are the most common, whereas those entering the small intestine meridian (21) are the least.

Fig. S2 Summary of CMM properties, pharmacological effects, and efficacies. (A) Distribution of CMM properties by herb count. (B) Distribution of CMM pharmacological effects by herb count. (C) Distribution of CMM efficacies by herb count.

## Normalization preserved the overall structure of the pharmacological effect–Chinese Materia Medica association matrix

Using the 60th percentile of all non-zero Chinese Materia Medica-wise proportional scores as the binarization threshold, the Q60 cutoff was 0.03226. This normalization strategy generated 4,229 positive Chinese Materia Medica–pharmacological effect associations, which was comparable to the 4,060 positive associations obtained using the original publication count ≥10 criterion.

The normalized matrix showed high consistency with the original binary matrix. At the pharmacological-effect level, the numbers of associated Chinese Materia Medica across the 40 pharmacological effects were highly correlated between the two methods, with a Pearson correlation coefficient of 0.981 (Fig S3A). At the individual Chinese Materia Medica–pharmacological effect cell level, 21,999 out of 24,640 labels remained unchanged, corresponding to a cell-wise agreement of 89.3% (Fig S3B).

These results indicate that herb-wise normalization mainly modified a subset of boundary associations, while the overall structure and pharmacological-effect coverage pattern of the dataset remained largely consistent with the original publication count–based construction strategy. Therefore, the potential influence of research-volume bias on the overall model construction was considered acceptable.

Fig S3. Robustness analysis of herb-wise Q60 normalization. (A) Pharmacological-effect coverage correlation between the original publication count ≥10 criterion and the Q60-normalized criterion. Each point represents one pharmacological effect. (B) Cell-wise agreement heatmap comparing the original and Q60-normalized binary matrices. Green indicates consistent labels and orange indicates inconsistent labels.

## BA restores lipid-metabolism-related gene expression in the HepG2 lipid-overload model

To address this concern, we selected two targets for RT-qPCR validation: FABP1, a key target within our efficacy-driven network, and CYP51A1, which was not included in the network but was selected as a lipid-metabolism-related downstream gene involved in cholesterol biosynthesis and sterol homeostasis (Fig. S4). In the oleic acid/palmitic acid-induced HepG2 model, the mRNA expression levels of both *FABP1* and *CYP51A1* were significantly reduced, whereas BA treatment markedly reversed these changes. Previous studies have also reported reduced *FABP1* expression in nonalcoholic fatty liver disease, suggesting that its dysregulation contribute to impaired hepatic fatty-acid handling and lipotoxicity. Therefore, the restoration of *FABP1* expression by BA is biologically consistent with the observed improvement in lipid accumulation[14].

*CYP51A1* encodes lanosterol 14α-demethylase, an important enzyme involved in cholesterol biosynthesis and the maintenance of sterol homeostasis. Previous studies in HepG2 cells have shown that *CYP51A1* knockout leads to marked accumulation of lanosterol and related sterol intermediates, accompanied by broad metabolic and transcriptional alterations[15]. Hepatocyte-specific *CYP51A1* knockout has also been reported to reduce cholesterol ester levels, promote sterol precursor accumulation, and induce hepatic inflammation and fibrosis[16]. In addition, downregulation of *CYP51A1* has been observed in a Western diet-induced hepatic steatosis model[17].

These results indicate that the BA-induced reduction in lipid accumulation was accompanied by partial restoration of fatty-acid- and sterol-metabolism-related molecular changes, providing preliminary target-level support for the computational predictions.

Fig S4. Effects of bornyl acetate on lipid-metabolism-related gene expression in the HepG2 lipid-overload model. (A) Relative mRNA expression levels of FABP1 (B) Relative mRNA expression levels of CYP51A1. Data are presented as mean ± SD. ^#^*P* < 0.05 versus the control group; **P* < 0.05 versus the model group.

## Algorithm frequencies and co-occurrence patterns revealed by AutoML

Frequency analysis of the algorithms revealedthat FSMIC (15), ClusterCentroids (15), and KNN (13) were the most frequently used methods for feature selection, resampling, and classification, respectively (Fig S5A). Correspondingly, their pairwise combinations—FSMIC–ClusterCentroids, FSMIC–KNN, and ClusterCentroids–KNN—were also the most frequent in their respective categories (Fig S5B). Among all algorithmic combinations, the triple combination FSMIC–ClusterCentroids–KNN occurred most frequently (n = 8), underscoring its dominant role in the modeling workflow.

Fig. S5. Algorithmic component evaluation. (A) Frequency distribution of individual algorithms. (B) Frequency distribution of pairwise algorithm combinations.

## Efficacy-guided pathway prioritization beyond the funnel effect

KEGG enrichment further showed that lipid-related pathways were preferentially ranked in the dissipating-dampness efficacy network compared with same-size random compound networks. Lipid and atherosclerosis, arachidonic acid metabolism, linoleic acid metabolism, and fluid shear stress and atherosclerosis all ranked within the top 20 pathways in the efficacy-driven network and showed better percentile rankings than the random-network average (Supplementary Fig. S6).

Fig. S6. KEGG enrichment and random-network comparison of lipid-related pathways in the dissipating-dampness efficacy network. (A) Top 20 KEGG pathways enriched in the efficacy network of NRR. (B) Percentile ranking comparison of the four lipid-related pathways between the efficacy-driven network and same-size random compound networks.

## Non-cytotoxic concentration screening

The CCK-8 assay (Fig S7) showed that *Notopterygii Rhizoma et Radix* extract (NRRE) was non-toxic at concentrations up to 312.5 μg/mL, and that 5-geranoxy-7-methoxycoumarin (GMOC), alloisoimperatorin (AIP), and acetyl borneol (BA) were non-toxic at concentrations up to 125 μM. These concentrations were therefore selected for subsequent cell treatment experiments.

Fig S7 Cell viability of *HepG2* cells treated with NRRE, AIP, BA, and GMOC. (A) Cell viability of HepG2 cells treated with NRRE at 19.53–625 μg/mL. (B) Cell viability of HepG2 cells treated with AIP at 7.81–250 μM. (C) Cell viability of HepG2 cells treated with BA at 7.81–250 μM. (D) Cell viability of HepG2 cells treated with GMOC at 7.81–250 μM.

# References

1.	Savander S, Curabaz NN, Abbasi AM, Khan A, Saeed K, Tanoli Z: **Data-driven strategies for drug repurposing: insights, recommendations, and case studies.** *Brief Bioinform* 2025, **26:**bbaf625.

2.	Zakharov AV, Peach ML, Sitzmann M, Nicklaus MC: **QSAR modeling of imbalanced high-throughput screening data in PubChem.** *J Chem Inf Model* 2014, **54:**705–712.

3.	Bie B, Sun J, Guo Y, Li J, Jiang W, Yang J, Huang C, Li Z: **Baicalein: A review of its anti-cancer effects and mechanisms in Hepatocellular Carcinoma.** *Biomedicine & Pharmacotherapy* 2017, **93:**1285–1291.

4.	Chen H, Gao Y, Wu J, Chen Y, Chen B, Hu J, Zhou J: **Exploring therapeutic potentials of baicalin and its aglycone baicalein for hematological malignancies.** *Cancer letters* 2014, **354:**5–11.

5.	Shen Y-C, Chiou W-F, Chou Y-C, Chen C-F: **Mechanisms in mediating the anti-inflammatory effects of baicalin and baicalein in human leukocytes.** *European journal of pharmacology* 2003, **465:**171–181.

6.	Li Y, Zhao J, Hölscher C: **Therapeutic potential of baicalein in Alzheimer’s disease and Parkinson’s disease.** *CNS drugs* 2017, **31:**639–652.

7.	Dermani FK, Samadi P, Rahmani G, Kohlan AK, Najafi R: **PD-1/PD-L1 immune checkpoint: Potential target for cancer therapy.** *Journal of Cellular Physiology* 2019, **234:**1313–1325.

8.	Gagliani N, Vesely MCA, Iseppon A, Brockmann L, Xu H, Palm NW, de Zoete MR, Licona-Limón P, Paiva RS, Ching T, et al: **TH17 cells transdifferentiate into regulatory T cells during resolution of inflammation.** *Nature* 2015, **523:**221–U225.

9.	Reddy VP, Aryal P, Soni P: **RAGE Inhibitors in Neurodegenerative Diseases.** *Biomedicines* 2023, **11**.

10.	Boccardi V, Mancinetti F, Mecocci P: **Oxidative Stress, Advanced Glycation End Products (AGEs), and Neurodegeneration in Alzheimer's Disease: A Metabolic Perspective.** *Antioxidants* 2025, **14**.

11.	Bannister K, Sachau J, Baron R, Dickenson AH: **Neuropathic Pain: Mechanism-Based Therapeutics.** In *Annual Review of Pharmacology and Toxicology, Vol 60.* *Volume* 60. Edited by Insel PA; 2020: 257–274: *Annual Review of Pharmacology and Toxicology*].

12.	Szatmary P, Grammatikopoulos T, Cai WH, Huang W, Mukherjee R, Halloran C, Beyer G, Sutton R: **Acute Pancreatitis: Diagnosis and Treatment.** *Drugs* 2022, **82:**1251–1276.

13.	Wang J, Zhou YM, Zhang HL, Hu LH, Liu JT, Wang L, Wang TY, Zhang HY, Cong LP, Wang Q: **Pathogenesis of allergic diseases and implications for therapeutic interventions.** *Signal Transduction and Targeted Therapy* 2023, **8**.

14.	Guzmán C, Benet M, Pisonero-Vaquero S, Moya M, García-Mediavilla MV, Martínez-Chantar ML, González-Gallego J, Castell JV, Sánchez-Campos S, Jover R: **The human liver fatty acid binding protein (FABP1) gene is activated by FOXA1 and PPARα; and repressed by C/EBPα: Implications in FABP1 down-regulation in nonalcoholic fatty liver disease.** *Biochimica et Biophysica Acta (BBA)-Molecular and Cell Biology of Lipids* 2013, **1831:**803–818.

15.	Skubic C, Trček H, Nassib P, Kreft T, Walakira A, Pohar K, Petek S, Režen T, Ihan A, Rozman D: **Knockouts of CYP51A1, DHCR24, or SC5D from cholesterol synthesis reveal pathways modulated by sterol intermediates.** *Iscience* 2024, **27**.

16.	Lorbek G, Perše M, Jeruc J, Juvan P, Gutierrez-Mariscal FM, Lewinska M, Gebhardt R, Keber R, Horvat S, Björkhem I: **Lessons from hepatocyte-specific Cyp51 knockout mice: impaired cholesterol synthesis leads to oval cell-driven liver injury.** *Sci Rep* 2015, **5:**8777.

17.	Roberts MD, Mobley CB, Toedebush RG, Heese AJ, Zhu C, Krieger AE, Cruthirds CL, Lockwood CM, Hofheins JC, Wiedmeyer CE: **Western diet-induced hepatic steatosis and alterations in the liver transcriptome in adult Brown-Norway rats.** *BMC Gastroenterol* 2015, **15:**151.
